# Phylogeographic diversification and postglacial range dynamics shed light on the conservation of the kelp *Saccharina japonica*

**DOI:** 10.1111/eva.12756

**Published:** 2019-01-12

**Authors:** Jie Zhang, Jianting Yao, Zi‐Min Hu, Alexander Jueterbock, Norishige Yotsukura, Tatiana N. Krupnova, Chikako Nagasato, Delin Duan

**Affiliations:** ^1^ Key Lab of Experimental Marine Biology, Institute of Oceanology Chinese Academy of Sciences Qingdao China; ^2^ Laboratory for Marine Biology and Biotechnology Qingdao National Laboratory for Marine Science and Technology Qingdao China; ^3^ Center for Ocean Mega‐Science Chinese Academy of Sciences Qingdao China; ^4^ Faculty of Biosciences and Aquaculture Nord University Bodø Norway; ^5^ Field Science Centre for Northern Biosphere Hokkaido University Sapporo Japan; ^6^ Pacific Research Fisheries Centre (TINRO‐Centre) Vladivostok Russia; ^7^ Muroran Marine Station, Field Science Center for Northern Biosphere Hokkaido University Muroran Japan

**Keywords:** glacial refugium, phylogeographic diversification, range dynamics, *Saccharina japonica*, secondary contact

## Abstract

Studies of postglacial range shifts could enhance our understanding of seaweed species’ responses to climate change and hence facilitate the conservation of natural resources. However, the distribution dynamics and phylogeographic diversification of the commercially and ecologically important kelp *Saccharina japonica* in the Northwest Pacific (NWP) are still poorly surveyed. In this study, we analyzed the evolutionary history of *S. japonica* using two mitochondrial markers and 24 nuclear microsatellites. A STRUCTURE analysis revealed two partially isolated lineages: lineage H, which is scattered along the coast of Japan; and lineage P, which occurs along the west coast of the Japan Sea. Ecological niche modeling projections to the Last Glacial Maximum (LGM) revealed that the southern coasts of the Japan Sea and the Pacific side of the Oshima and Honshu Peninsulas provided the most suitable habitats for *S. japonica*, implying that these regions served as ancient refugia during the LGM. Ancient isolation in different refugia may explain the observed divergence between lineages P and H. An approximate Bayesian computation analysis indicated that the two lineages experienced post‐LGM range expansion and that postglacial secondary contact occurred in Sakhalin. Model projections into the year 2,100 predicted that *S. japonica* will shift northwards and lose its genetic diversity center on the Oshima Peninsula in Hokkaido and Shimokita Peninsula in Honshu. The range shifts and evolutionary history of *S. japonica* improve our understanding of how climate change impacted the distribution range and diversity of this species and provide useful information for the conservation of natural resources under ongoing environmental change in the NWP.

## 1 INTRODUCTION

Climate changes during the late Pleistocene glaciations influenced the present‐day distribution and phylogeographic structure of marine flora and fauna (e.g., Coyer, Peters, Stam, & Olsen, 2003; Hansen, Mensberg, & Berg, 1999; Liu, Gao, Wu, & Zhang, 2007; Reusch, Stam, & Olsen, 2000). Temperate species commonly responded to the alternation between glacial and interglacial periods with range contractions and expansions (Jim & Bennett, 2008). Some lineages were able to survive in refugia and expand northward as temperatures warmed (Hewitt, 1999, 2000). Refugia with long‐term population persistence often display high genetic diversity and unique gene variations (Hewitt, 2000). Identifying the locations of refugia and postglacial recolonization pathways provides valuable information for conserving local genetic variation and evolutionary integrity.

Numerous species worldwide respond to modern climate change with poleward range shifts (Davis & Shaw, 2001; Parmesan & Yohe, 2003; Rosenzweig et al., 2008), and ample evidence has shown that modern climate change reduces the range of seaweeds and leads to decline in natural resources (Assis et al., 2017; Fernandez, 2011; Smale & Wernberg, 2013; Wernberg, Russell, Thomsen et al., 2011). Poleward range shifts and trailing edge contractions are common responses to increasing temperatures for temperate seaweeds (e.g., Jueterbock et al., 2013; Müller, Laepple, Bartsch, & Wiencke, 2009; Neiva, Assis, Fernandes, Pearson, & Serrao, 2014). Thus, predicting the range shift and habitat loss at the rear edge are essential for the conservation and management of seaweeds.

The Northwest Pacific (NWP) is particularly rich in marine flora and fauna, including large assemblages of endemic algae (Kerswell, 2006). This area experienced repeated glacial oscillations and is characterized by complex ocean current systems that influence the distributions and evolutionary processes of seaweeds, such as *Chondrus ocellatus*, *Sargassum fusiforme, S. horneri*, *S. hemiphyllum, *and *S. thunbergii *(Cheang, Chu, & Ang, 2010; Hu et al., 2011, 2017, 2015; Hu, Zhang, Lopez‐Bautista, & Duan, 2013; Li et al., 2017). Recent phylogeographic studies have focused more on the population genetic diversity and demographic history of seaweeds in the NWP (Hu et al., 2017; Kantachumpoo, Uwai, Noiraksar, & Komatsu, 2014; Kim, Hoarau, & Boo, 2012; Uwai, Emura, Morita, Kurashima, & Kawai, 2009; Yotsukura, Maeda, Abe, Nakaoka, & Kawai, 2016), and few reports have investigated the glacial refugia and range dynamics of seaweeds in this region (Cheang, Chu, & Ang, 2008; Coyer, Hoarau, Van Schaik, Luijckx, & Olsen, 2011; Lee et al., 2012).

*Saccharina japonica *is a cold‐temperature brown seaweed that is native to northern Japan and Far Eastern Russia (Supporting Information Figure S1), and it was introduced to northern China and South Korea in the early 1920s and 1970s, respectively (Hwang, Ha, & Park, 2018; Tseng & Wu, 1962). This kelp is endemic and dominant in the north of the Japan Sea and provides an ideal model to analyze how the climate changes have influenced the genetic diversity and postglacial range shifts of kelps in this region. The Japan Sea spans approximately 1 × 10^6^ km^2^ and is a semi‐marginal sea that differs from other marginal seas by its four shallow sills (the depth of Tartar Strait and Soya Strait <50 m) (Supporting Information Figure S1). During the Last Glacial Maximum (LGM) (*c*. 18–21 kya), the sea levels in the Japan Sea were *c*. 120 m lower than they are today, and the Japan Sea was nearly isolated from the Pacific Ocean (Oba et al., 1991; Wang, 1999). Even the sill depths are very close to that of the LGM sea‐level lowstand, and two of the four connecting straits of the basin were deep enough to maintain limited water exchange with the Pacific Ocean (Tsugaru Strait, sill depth 116 m) and East China Sea (Tsushima Strait, sill depth 131 m) (Wang, 1999). Although a continental ice cap did not develop in the Japan Sea during the LGM (Frenzel, Pecsi, & Velichko, 1992), the opening of these four straits influenced by the sea‐level fluctuation could have had a significant impact on the present‐day distribution patterns of marine organisms along the coasts of the Japan Sea (Azuma, Zaslavskaya, Yamazaki, Nobetsu, & Chiba, 2017; Canino, Spies, Cunningham, Hauser, & Grant, 2010; Cheang et al., 2008; Hiruta, Ikoma, Katoh, Kajihara, & Dick, 2017). Glacial vicariance resulted in the phylogeographic diversification of kelp populations (Neiva et al., 2018); however, the effects of the post‐LGM opening of the four straits along the Japan Sea on the distribution of *S. japonica* are poorly understood. Two shallow genealogies have been previously reported in *S. japonica*, and they were explained by niche differentiation (Zhang et al., 2015). However, genetic admixture would be expected when genetically differentiated populations reach secondary contact zones (e.g., seaweed, Hu et al., 2011; seagrass, Alberto et al., 2010). Several reports have identified locations of refugia, such as in the Okinawa Trough, around the Jeju Island and to the southwest of the Japanese Archipelago (Cheang et al., 2008; Hu et al., 2015; Lee et al., 2012). *S. japonica* may have survived in northern glacial refugia during the LGM considering the restricted distribution range of wild indigenous populations in the NWP.

Without fossil records, tracing the evolutionary history of extant seaweeds is difficult. Nevertheless, genetic data (mitochondrial DNA and microsatellite loci) combined with ecological niche models (ENMs) allow us to infer lineage diversity and postglacial range dynamics. The overall aim of this study was to improve our understanding of how *S*. *japonica* responded to climate change during the LGM, and to infer management and conservation strategies for this species under ongoing climate change. More particularly, our objectives were to (a) identify potential glacial refugia and subsequent recolonization routes; (b) investigate how the opening of the four straits along the Japan Sea influenced the distribution patterns and secondary contact; and (c) assess how climate change affected the range shift of *S. japonica.*

## 2 MATERIALS AND METHODS

### 2.1 Sampling and DNA extraction

Between 2011 and 2013, we sampled 20 populations along the NWP coast from Sakhalin, Russia (48°50′N, 141°55′E) to Gangneung, South Korea (37°47′N, 128°55′E) (Zhang et al., 2015). Between 2013 and 2014, we sampled 15 additional populations (5 populations from Primorsky, Russia; 7 populations from Hokkaido, Japan; 3 populations from Honshu, Japan). Detailed sampling information is given in Supporting Information Table S1. Total genomic DNA was extracted from *S. japonica *using a plant genomic DNA kit (Tiangen, Beijing) according to the manufacturer's instructions.

### 2.2 Mitochondrial DNA (mtDNA) data sequencing and microsatellite genotyping

The mitochondrial protein‐coding *COI* and mitochondrial ribosomal *trn*W‐L were selected for genetic analysis (Balakirev, Krupnova, & Ayala, 2012; Zhang et al., 2015). The PCR procedures were conducted as in Zhang et al. (2015). The obtained sequences were proofed and aligned in bioedit v. 7.1 (Hall, 1999) and geneious v. 6.0.5 (Biomatters, New Zealand, http://www.geneious.com).

In total, 768 individuals from 35 populations were genotyped at 24 genome‐wide microsatellite loci (Li, Zhang, Yao, Wang, & Duan, 2015) with three multiplexing sets. The microsatellite marker characteristics are listed in Supporting Information Table S2. PCR was performed according to Zhang et al. (2017).

### 2.3 Genetic statistics for mtDNA and microsatellite markers

For mitochondrial markers, a total length of 1890 bp was applied after combining the protein‐coding *COI* and ribosomal *trn*W‐L. We calculated the number of segregating sites (*S*), number of haplotypes (*h*), haplotype diversity (Hd), and nucleotide diversity (Pi) with dnasp v.5 (Librado & Rozas, 2009). A median‐joining network was constructed using network v.4.5.1.6 based on the concatenated sequences (Bandelt, Forster, & Rohl, 1999).

For microsatellites, scoring errors and null alleles were corrected with micro‐checker v.2.2 (Van Oosterhout, Hutchinson, Wills, & Shipley, 2004). Tests of linkage disequilibrium were conducted for each population and each locus in genepop v.4.1 (Rousset, 2008), and significance levels were adjusted for multiple comparisons following the standard Bonferroni correction (Rice, 1989). Allelic richness was estimated in fstat v.2.9.3 (Goudet, 1995). The number of alleles observed (*N*_A_), number of private alleles (*N*_P_), and observed and expected heterozygosities (*H*_O_ and *H*_E_, respectively) were calculated using genalex v.6.41 (Peakall & Smouse, 2006).

### 2.4 Genetic structure

We estimated the population genetic structure using two different Bayesian clustering methods in structure v.2.3.3 (Pritchard, Stephens, & Donnelly, 2000) and baps v.6.0 (Cheng, Connor, Sirén, Aanensen, & Corander, 2013). For the microsatellites, ten independent analyses were performed for *K* = 1–10 based on 10^6^ Markov chain Monte Carlo iterations following a burn‐in period of 10^5^ steps. An admixture model was used after assuming correlated allele frequencies among the populations. The best *K* value was determined by the mean log‐likelihood of the data (ln *P*(*X*|*K*)) and the Delta *K* (Δ*K*) method (Evanno, Regnaut, & Goudet, 2005) in structure harvester (Earl & Vonholdt, 2012). The final results were summarized in clumpp v.1.1.2 (Jakobsson & Rosenberg, 2007) and displayed with distruct v.1.1 (Rosenberg, 2004). We also used spatial clustering of groups to infer the population structure in *S. japonica *with baps v.6.0 based on concatenated mitochondrial DNA, and 10 runs (*K* = 20) were performed to ensure the convergence and consistency of the results.

Population genetic differentiation was estimated by calculating the *F*_ST_ in arlequin v.3.5 (Excoffier & Lischer, 2010). The significance of the *F*_ST_ value was tested by 10^4^ permutations for each pairwise comparison. A hierarchical molecular variance analysis (AMOVA) was conducted to partition the genetic variance using arlequin v.3.5.

### 2.5 Approximate Bayesian computation (ABC) analyses

**Figure 1:**
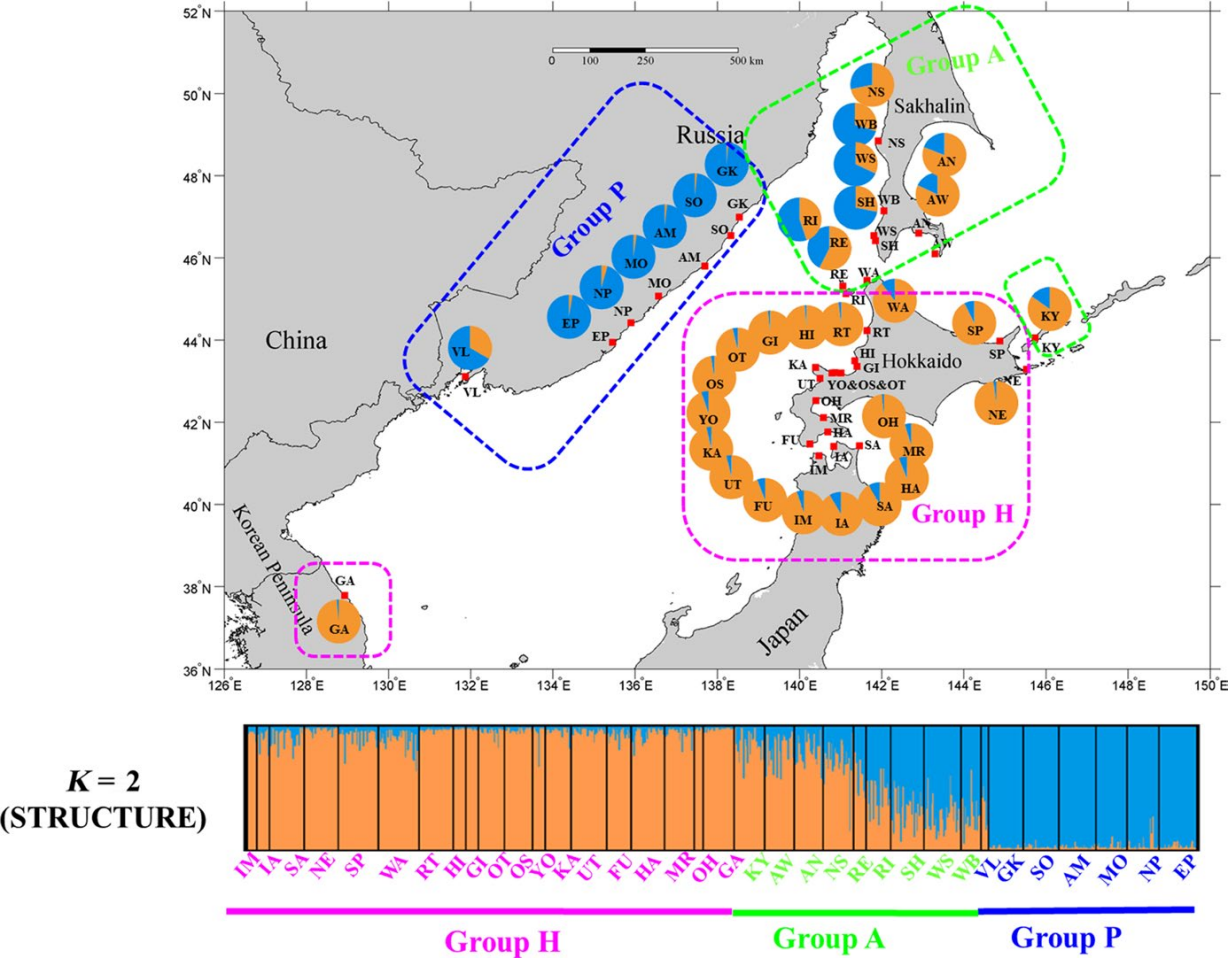
Geographic distribution of three genetic groups based on STUCTURE (*K* = 2) analysis

Evolutionary scenarios since the LGM were compared in diyabc v.2.1.0 (Cornuet et al., 2014) based on the 24 microsatellites and the combined microsatellites and two mitochondrial loci data. We confirmed that the main results were basically unchanged regardless of sample size and sample selection in the preliminary runs. To reduce computational complexity, we randomly selected 352 individuals from 16 populations for the following DIYABC analyses. All scenarios were defined based on three major groups identified by the STRUCTURE analyses (Figure 1): (a) Group H consisted of 8 populations (RT, HI, YO, UT, FU, SA, HA, and OH) from Hokkaido and Honshu (assignment coefficient >90%), (b) group P contained three populations (NP, AM, and GK) from the west coast of the Japan Sea (Primorsky Krai, Russia) (assignment coefficient >90%), and (c) group A included admixed populations (SH, NS, AW, WA, and RE) from Sakhalin, Russia (assignment coefficient <90%). We excluded the non‐native GA population from our ABC analyses because it was introduced by human activity in the 1970s (Hwang et al., 2018). We also excluded population VL from the west coast of the Japan Sea due to the small sample size (<10 individuals). We conducted a DIYABC analysis with two stages as follows.

**Figure 2:**
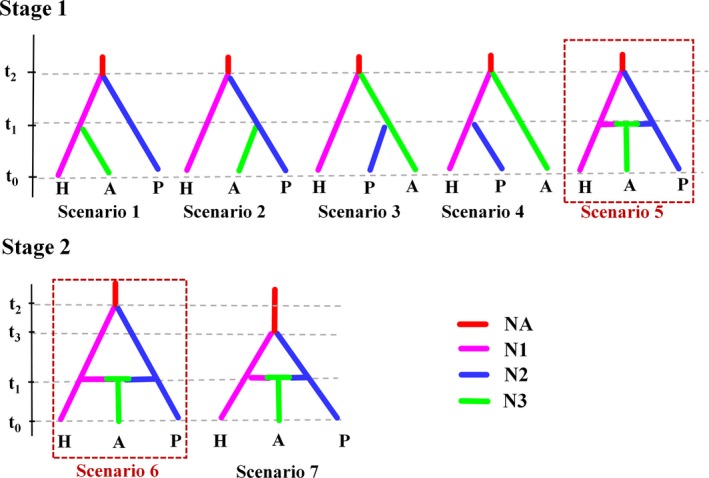
Probable demographic scenarios tested with approximate Bayesian computation as implemented in DIYABC. For parameters, descriptions, and priors, see Supporting Information Table S3

In Stage 1, we defined 5 broad‐scale scenarios (Scenarios 1–5, Figure 2) to test whether genetic admixture occurs during the postglacial colonization. The prior values of the effective population size, time, and admixture rate for all cases in Stage 1 are listed in Supporting Information Table S3. Other prior values for some parameters were kept at default settings. Because DIYABC requires a population to be traced back as an ancestral one, we set NA as the ancestor. For each scenario, we placed the current populations at time *t*_0_ and divergence from the most recent common ancestral population (MRCAP) at *t*_2_ (Figure 2).

Scenario 1: *Hierarchical split model*: Group H merged with admixed group A at *t*_1_ and then with group P at *t*_2_.
Scenario 2: *Hierarchical split model*: Group P merged with admixed group A at *t*_1 _and then with group H at *t*_2_.
Scenario 3: *Hierarchical split model*: The admixed group A merged with group P at *t*_1_ and then with group H at *t*_2_.
Scenario 4: *Hierarchical split model*: Group H merged with group P at *t*_1_ and then with the admixed group A at *t*_2_.
Scenario 5: *Isolation with admixture model:* Group A was created by admixture of group H and group P at *t*_1_ and then group H merged with group P at *t*_2_.

In Stage 2, we tested two fine‐scale models to determine whether genetic divergence between the H and P lineages occurred pre‐ or post‐LGM based on the best model derived from Stage 1 (Figure 2). These scenarios are detailed as follows: (a) scenario 6 considered that the genetic divergence between H and P lineages occurs pre‐LGM; (b) scenario 7 considered that H and P lineages diverged from each other at post‐LGM. The prior values of the parameters for scenario 6 and scenario 7 are listed in Supporting Information Table S3.

We created a reference table consisting of 10^6^ simulated data sets per scenario. Only 1% of the simulated data sets closest to the observed data were adopted to estimate the relative posterior probability of each scenario with the logistic regression method (Cornuet et al., 2008). We calculated the posterior predictive error over 500 data sets to evaluate the level of confidence in the scenario choice. The mean number of alleles, mean gene diversity, and mean allele size variance across loci were chosen for single sample statistics. In addition, the* F*_ST_ values, mean index of classification, and (δμ)^2^ distance were chosen for two sample statistics.

### 2.6 Ecological niche modeling

Model selection and the habitat suitability projection analysis were conducted using three files encompassing the occurrence records, background points, and environmental variables. The 49 *S. japonica* occurrence records were based on field observations, previous studies, and online databases (Supporting Information Table S4). To ensure similar record density throughout the species’ range (Phillips et al., 2009), we used the java program “Occurencethinner” v.1.01 (Verbruggen, 2012) and the r package “KernSmoth” v.2.23 (Wand, 2014) to remove 25 occurrence sites from areas with high local densities. The environmental conditions in the distributional range of this kelp were captured by 1,000 randomly chosen backgrounds points (also referred to as pseudo‐absence locations) within 20° to 58° latitude and 110° to 160° longitude using the r package “raster” (Hijmans, 2015) (Supporting Information Figure S2). In combination with the occurrence locations, the background points allow to discriminate suitable from unsuitable environmental conditions. Current and future environmental variables were downloaded from the Bio‐ORACLE database (http://www.oracle.urgent.be/index.html, real values). All 23 geophysical, biotic, and climate variables provided in the original bio‐oracle v.1.0 dataset (Tyberghein et al., 2012), and the 6 ice cover derivatives provided in the extended set (bio‐oracle v.2.0) (Assis et al., 2018) were considered as potentially important environmental variables. Environmental variables for the LGM were derived from PaleoMARSPEC data layers (Sbrocco & Barber, 2013). The habitat suitability for *S. japonica *under past, present‐day, and future environmental conditions was estimated using correlative ENMs compiled with the program maxent v.3.3.3e (Phillips & Dudik, 2008). To avoid overfitting the maxent models to the occurrence records, we reduced the set of environmental variables in a stepwise fashion with the r package “MaxentVariableSelection” (Jueterbock, Smolina, Coyer, & Hoarau, 2016) by excluding variables with a relative contribution score <5% or a correlation of >0.9 with other variables (Supporting Information Table S5).

## 3 RESULTS

### 3.1 Population genetic characteristics

The MICRO‐CHECKER analysis did not identify scoring errors or large allele dropouts, although it did find evidence for null alleles (1.36% of population‐by‐locus pairs). However, null alleles were not overrepresented on any particular locus or populations. No deviations from Hardy–Weinberg equilibrium (HWE) were observed in the populations or loci. Linkage disequilibrium was not detected between pairs of microsatellite loci after Bonferroni sequential corrections (adjusted *p* = 0.0002) (Rice, 1989). The polymorphism information content (*PIC*) of the microsatellite loci ranged from 0.34 to 0.94 (Supporting Information Table S2), and a total of 6–59 alleles were identified in all kelp populations. The allelic richness per locus and population based on a minimum sample size of 6 diploid individuals ranged from 2.61 to 9.01 (Supporting Information Table S2). Ultimately, we used all 24 microsatellites for all subsequent analyses.

The genetic diversity based on mtDNA indicated that population HA (Oshima Peninsula, Hokkaido, Japan) had the highest nucleotide diversity (Pi = 0.00158) and population SA (Shimokita Peninsula, Honshu, Japan) had the highest haplotype diversity (Hd = 0.81) (Table 1). For the microsatellites, the top three highest genetic diversity values were detected in FU (*H*_O_: 1.61; *H*_E_: 0.62; *A*_R_: 5.17) and HA (*H*_O_: 1.59; *H*_E_: 0.68; *A*_R_: 4.99) in the Oshima Peninsula, Hokkaido, and in SA in the Shimokita Peninsula, Honshu (*H*_O_: 1.55; *H*_E_: 0.60; *A*_R_: 4.89). All estimates showed that three populations (FU, HA, and SA) from the Oshima Peninsula and the Shimokita Peninsula had higher diversities (*H*_O_: 1.55–1.61; *H*_E_: 0.60–0.68; *A*_R_: 4.89–5.17) than other populations (*H*_O_: 0.58–1.47; *H*_E_: 0.24–0.65; *A*_R_: 2.30–4.76; Table 1).

**Table 1:** Molecular diversity estimates for 35 populations of *Saccharina japonica*

Code	mtDNA					Microsatellites					
	*N*	*S*	*h*	Hd	Pi (10^−3^)	*N*	*N*_A_	*N*_P_	*H*_O_	*H*_E_	*A_R_*
Shimokita Peninsula of Honshu, Japan											
IM	8	2	2	0.57	0.60	8	5.13 (0.56)	0.08 (0.06)	1.27 (0.12)	0.53 (0.06)	4.56 (0.46)
IA	10	3	3	0.60	0.60	10	5.92 (0.62)	0.08 (0.06)	1.32 (0.13)	0.57 (0.06)	4.66 (0.44)
SA	27	9	9	0.81	1.04	28	8.83 (1.12)	0.25 (0.09)	1.55 (0.14)	0.60 (0.05)	4.89 (0.43)
Oshima Peninsula of Hokkaido, Japan											
FU	19	4	5	0.58	0.41	20	8.17 (1.05)	0.21 (0.13)	1.61 (0.13)	0.62 (0.04)	5.17 (0.42)
HA	28	9	6	0.71	1.58	27	8.46 (0.82)	0.38 (0.15)	1.59 (0.11)	0.68 (0.04)	4.99 (0.33)
MR	26	3	4	0.40	0.25	24	7.13 (0.88)	0.17 (0.10)	1.28 (0.13)	0.54 (0.04)	4.14 (0.37)
OH	7	3	3	0.76	0.81	7	4.63 (0.47)	0.04 (0.04)	1.19 (0.11)	0.65 (0.05)	4.32 (0.41)
Hokkaido, Japan (excluding Oshima Peninsula)											
NE	28	1	2	0.51	0.27	28	3.92 (0.73)	0.13 (0.07)	0.65 (0.13)	0.29 (0.05)	2.50 (0.34)
SP	32	2	3	0.56	0.42	32	5.04 (0.65)	0.08 (0.06)	0.97 (0.13)	0.47 (0.05)	3.28 (0.34)
WA	33	9	8	0.51	0.52	33	5.58 (0.82)	0.13 (0.09)	0.97 (0.14)	0.44 (0.05)	3.25 (0.38)
RE	10	4	3	0.38	0.42	10	4.42 (0.57)	0.08 (0.06)	0.99 (0.13)	0.48 (0.06)	3.66 (0.43)
RI	20	0	1	0.00	0.00	20	5.17 (0.71)	0.00 (0.00)	1.02 (0.14)	0.48 (0.06)	3.46 (0.39)
RT	28	2	3	0.31	0.17	28	6.00 (0.94)	0.17 (0.10)	1.01 (0.14)	0.45 (0.05)	3.40 (0.39)
HI	10	1	2	0.47	0.25	10	4.13 (0.54)	0.04 (0.04)	0.96 (0.12)	0.49 (0.06)	3.42 (0.39)
GI	9	2	3	0.42	0.24	10	4.08 (0.60)	0.04 (0.04)	0.93 (0.12)	0.48 (0.06)	3.36 (0.42)
OT	21	2	3	0.34	0.19	21	6.75 (0.81)	0.33 (0.18)	1.32 (0.11)	0.55 (0.04)	4.29 (0.34)
OS	24	3	3	0.49	0.50	23	7.33 (0.94)	0.08 (0.06)	1.38 (0.15)	0.55 (0.06)	4.55 (0.43)
YO	10	4	4	0.64	0.59	10	5.83 (0.67)	0.00 (0.00)	1.38 (0.13)	0.59 (0.05)	4.76 (0.46)
KA	22	2	3	0.18	0.10	21	6.92 (0.86)	0.13 (0.07)	1.39 (0.13)	0.60 (0.04)	4.49 (0.40)
UT	28	3	4	0.21	0.11	29	7.42 (0.75)	0.08 (0.06)	1.38 (0.10)	0.57 (0.04)	4.26 (0.31)
Sakalin island, Russia											
KY	28	28	4	0.21	0.11	25	5.50 (0.82)	0.04 (0.04)	1.06 (0.13)	0.43 (0.05)	3.47 (0.38)
AW	24	3	4	0.24	0.13	24	5.71 (1.13)	0.13 (0.07)	0.98 (0.16)	0.42 (0.05)	3.34 (0.46)
AN	29	2	3	0.20	0.11	23	5.38 (0.75)	0.00 (0.00)	1.01 (0.13)	0.47 (0.06)	3.34 (0.38)
SH	27	4	5	0.40	0.26	27	6.17 (0.82)	0.13 (0.09)	0.99 (0.13)	0.45 (0.05)	3.35 (0.37)
WS	30	4	4	0.40	0.30	30	5.67 (0.82)	0.13 (0.09)	0.94 (0.13)	0.43 (0.05)	3.18 (0.36)
WB	16	2	3	0.24	0.13	16	4.58 (0.64)	0.04 (0.04)	0.89 (0.13)	0.41 (0.06)	3.16 (0.38)
NS	26	3	3	0.15	0.12	25	5.17 (0.94)	0.04 (0.04)	0.96 (0.14)	0.41 (0.05)	3.24 (0.42)
Primorsky, Russia											
GK	26	5	6	0.35	0.24	28	3.58 (0.53)	0.08 (0.06)	0.58 (0.12)	0.29 (0.05)	2.30 (0.29)
SO	27	5	4	0.28	0.27	29	3.79 (0.63)	0.04 (0.04)	0.59 (0.13)	0.29 (0.06)	2.36 (0.32)
AM	30	1	2	0.07	0.04	30	3.96 (0.66)	0.00 (0.00)	0.60 (0.12)	0.28 (0.06)	2.36 (0.32)
MO	25	7	8	0.80	0.66	25	4.04 (0.54)	0.04 (0.04)	0.59 (0.12)	0.27 (0.05)	2.43 (0.31)
NP	24	3	4	0.68	0.44	26	4.25 (0.55)	0.08 (0.06)	0.63 (0.12)	0.24 (0.05)	2.51 (0.30)
EP	30	0	1	0.00	0.00	30	4.29 (0.62)	0.21 (0.08)	0.63 (0.13)	0.29 (0.06)	2.54 (0.32)
VL	6	2	2	0.60	0.63	6	2.33 (0.21)	0.00 (0.00)	0.58 (0.09)	0.34 (0.06)	2.33 (0.21)
South Korea											
GA	27	5	4	0.33	0.27	25	6.88 (0.61)	0.13 (0.07)	1.47 (0.11)	0.58 (0.05)	4.63 (0.32)

Note

For mtDNA data: *h*: number of haplotypes; Hd: haplotype diversity; *N*: number of sequences analyzed; Pi: nucleotide diversity; *S*: number of segregating sites. For microsatellite data (24 loci):* A*_R_: allelic richness (allelic richness per locus and population based on minimum sample size of 6 diploid individuals); *H*_E_: expected heterozygosity; *H*_O_: observed heterozygosity; *N*: number of individuals analyzed; *N*_A_: number of alleles; *N*_P_: number of private alleles and their respective standard deviation (*SD*) in brackets. Sample codes are as in Table 1.

We detected 78 mitochondrial haplotypes from 765 mtDNA sequences in 35 populations (Supporting Information Figure S3; GenBank: KT963093‐KT963144, MK227354‐MK227387). In total, 16 haplotypes were shared among at least two populations, and the remaining 62 haplotypes were unique to a single population. The most frequent haplotype, H12, was shared by 311 individuals and accounted for 40.65% of all samples. Moreover, H54, which accounted for 23.27% of all samples, was mainly observed in the samples from Far Eastern Russia (Supporting Information Table S6). The median‐joining haplotype network exhibited a star‐like structure and revealed that the *S. japonica* population might experience demographic expansion (Supporting Information Figure S4). Moreover, 34 haplotypes were definable with a single mutation difference from H12, and 17 haplotypes were connected to H54 with one mutation step.

### 3.2 Population structure

The Evanno method and lnP (D) plots showed that the most likely number of genetic groups was *K* = 2 (Supporting Information Figure S5), which provided evidence of two genetic groups (group H and group P) and assigned a 90% probability of individuals to each of these groups (Figure 1). Group H consisted of individuals from Japan and South Korea, while group P contained individuals from the west coast of the Japan Sea. However, populations in Sakhalin (group A) had low assignment probabilities (<90%) to groups H and P (Figure 1).

Bayesian analyses of population structure using concatenated mtDNA sequences detected four genetic clusters: cluster 1 included populations in Hokkaido, Korea, and the east coast of Sakhalin; cluster 2 was observed only on the northwestern coast of Hokkaido; cluster 3 consisted of populations around Honshu Island; and cluster 4 contained most of the populations from the west coast of the Japan Sea and the west coast of Sakhalin (Supporting Information Figure S6). Although the mtDNA and microsatellites revealed an incongruent population structure in *S. japonica*, they also indicated that the populations on the west coast of the Japan Sea (group P and cluster 4) formed a diverged genetic lineage.

The microsatellite analysis revealed deep divergence between the populations on the west coast of the Japan Sea and those in Hokkaido (*F*_ST_ > 0.2; *p* < 0.001) (Supporting Information Table S7). Only 1% of the microsatellite‐based *F*_ST_ values exceeded 0.5, and 60% of the pairwise *F*_ST_ values based on mitochondrial markers exceeded 0.5 (Supporting Information Tables S7 and S8). Nevertheless, both markers indicated significant genetic differentiation between the Japanese and Russian populations. The AMOVA of the mitochondrial DNA indicated that the majority of variation was partitioned among four BAPS groups (51.13%), with only 16.5% of the variation among the two STRUCTURE groups with microsatellites (Supporting Information Tables S9).

### 3.3 Testing evolutionary scenarios using ABC

In the ABC simulations, both the SSR data and combined SSR and mitochondrial DNA data exhibited similar results; thus, only the simulation results for the combined data are shown. Prior distributions of parameters for each scenario in Stage 1 and Stage 2 are given in Supporting Information Table S3. A principle component analysis of the summary statistics for all prior scenario values showed that the simulated data were well‐fit to the observed data (Supporting Information Figures S7a and S8a). In Stage 1, the posterior probabilities based on a logistic regression for each scenario supported scenario 5 (admixture model) based on combined data (0.8981, 95% CI: 0.8322–0.9639) (Supporting Information Figure S9a) and SSR data (0.7828, 95% CI: 0.6819–0.8837) (Supporting Information Figure S9b). For scenario 5, the median effective population sizes values were 64,000 (95% CI: 38,400–91,800) for group H; 7,230 (95% CI: 4,870–9,390) for group P; and 35,400 (95% CI: 15,500–73,500) for group A (Supporting Information Table S10; Figure S7c). Scenario 5 (isolation with admixture model) assumed that the ancient populations (NA) diverged into groups H and P, and then, these two diverged lineages experienced secondary contact to form group A. The 95% confidence intervals for this scenario did not overlap with those obtained from the other four scenarios (scenario 1–4). In Stage 2, we obtained substantial support for scenario 6 based on both the combined data (0.6879, 95% CI: 0.6749–0.7009) and SSR data (0.7964, 95% CI: 0.7862–0.8066) (Supporting Information Figure S9c,d). This scenario indicated that the ancient group experienced pre‐LGM divergence and formed groups H and P. Subsequently, these two isolated lineages (H and P) experienced demographic expansion and had secondary contact in Sakhalin to form group A. For scenario 6, the effective population size was 3,310 (95% CI: 488–8,940) before the demographic expansion, and the median effective population size values were 63,600 (95% CI: 39,000–91,400) for group H; 6,910 (95% CI: 4,780–9,080) for group P; and 34,500 (95% CI: 15,400–73,200) for group A (Supporting Information Table S10; Figure S8c). The median value of the divergence time (*t*_2_) was 13,500 (95% CI: 10,800–18,900) generations, which corresponded to 27,000 years BP (Supporting Information Table S10; Figure S8c). The median value of the admixture time (*t*_1_) was 3,590 (95% CI: 2,970–9,550) generations, which corresponded to 7,180 years BP (Supporting Information Table S10; Figure S8c). The evaluation of confidence in scenario 6 revealed that the posterior predictive error computed over 500 data sets was 0.175, suggesting that the selected model was reliable. The principal component analyses showed that the observed data point was centered around the cluster of points for the simulated data based on the posterior distributions (Supporting Information Figure S8b), suggesting that scenario 6 was well‐fit to the observed data.

### 3.4 Ecological niche modeling

The model with the lowest AIC was built based on the following three uncorrelated environmental variables with a model contribution >12.19% (Supporting Information Figure S10; Table S5): sea surface temperature (SSTmean), maximum chlorophyll (Chlomax), and mean chlorophyll (Chlomean). The mean sea surface temperature was the most important variable (55% model contribution, Supporting Information Table S5) in discriminating suitable from nonsuitable habitats. Moreover, the chlorophyll maximum concentration had a contribution of 12.19%, while the chlorophyll mean concentration had a contribution of 32.79% (Supporting Information Table S5). The results showed that the chlorophyll concentration was negatively correlated with kelp habitat suitability (Supporting Information Figure S10), which may be due to improved light penetration at low chlorophyll (phytoplankton) levels.

**Figure 3:**
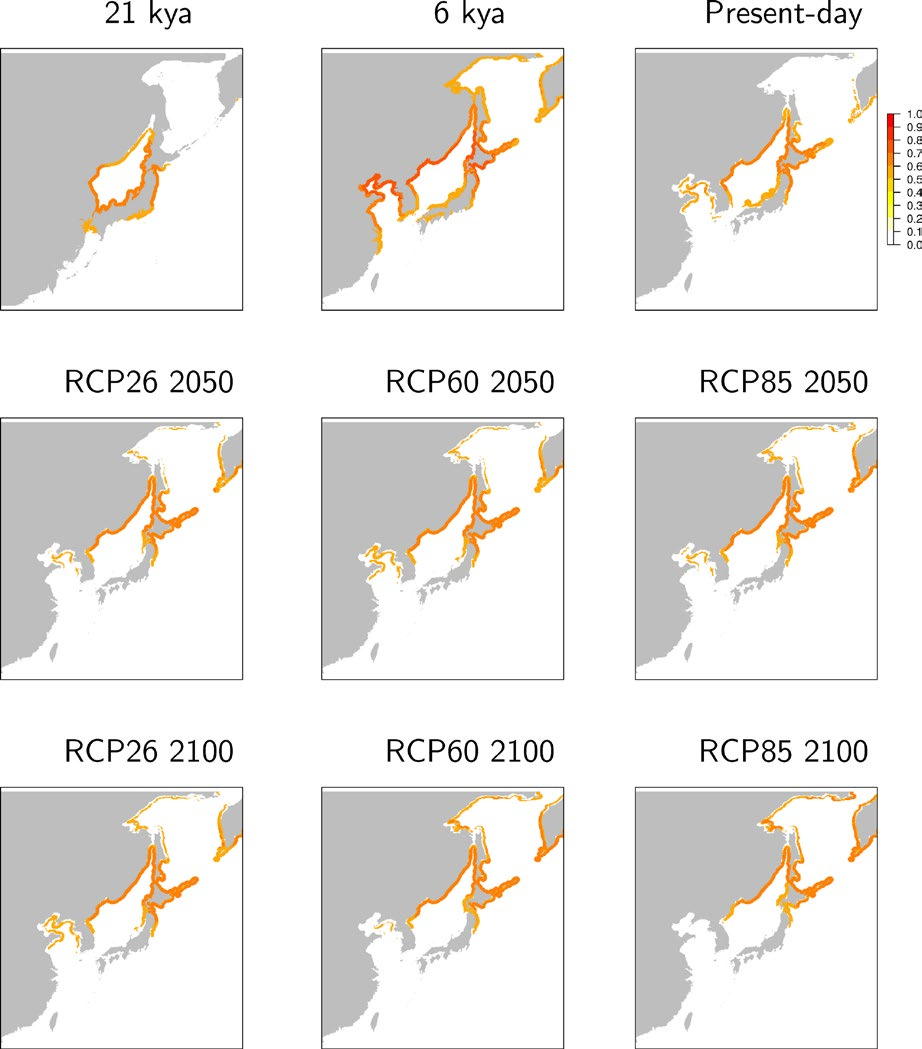
Range projections for *Saccharina japonica *for the Last Glacial Maximum (LGM), Mid‐Holocene (MH), present time and future (2050s and 2100s) with different climate scenarios (RCP26, RCP60, and RCP85)

The present‐day model (mean AUC = 0.85) was consistent with the species’ current distribution (Supporting Information Figures S11 and S12). The ENM predictions to the LGM reflected drastic reductions in suitable habitat, and the kelp populations may have only been present on the south coast of the Japan Sea and the east coast of Honshu Island during the LGM (Figure 3). In the mid‐Holocene (6 Kya), the melting of ice and opening of the Japan Sea allowed the kelp population to expand and colonize the north coast of the Japan Sea, the Korean Peninsula, northern China, and the Okhotsk Sea (Figure 3). The future projections indicate that this kelp will find new suitable habitat in the north and lose suitable habitat on the east coast of Hokkaido and the west coast of Honshu in Japan, particularly for the long‐term projection, which shows increasing greenhouse gas emissions over time (RCP 85; Figure 3).

## 4 DISCUSSION

### 4.1 Ancient isolation in glacial refugia as a driver for lineage divergence

Within a species, disjunct distributions of divergent genetic lineages reflect the occurrence of vicariant processes (Neiva, Pearson, Valero, & Serrao, 2012). Fragmentation and isolation of habitat during Pleistocene glaciations are typically considered the main reasons underlying the present lineage divergence for a variety of seaweeds, including *Durvillaea*, *Chondrus*, *Sargassum*, and *Saccharina *(Hu et al., 2011, 2015; Neiva et al., 2018; Weber, Edgar, Banks, Waters, & Fraser, 2017). During the LGM, the Japan Sea became almost isolated from the open ocean because the sea level dropped by approximately 120 m (Oba et al., 1991; Wang, 1999). Distribution models for the LGM projected that the most suitable habitat for *S. japonica* would occur along the southern Japan Sea (*c*. 35–44°N) and the Pacific coastlines of the Oshima Peninsula (Hokkaido) and the Shimokita Peninsula (Honshu) (Figure 3), and this distribution favored the occurrence of two refugia for surviving *S. japonica* populations. During that period, most populations in the Japan Sea were connected and may have formed a single unit/metapopulation (ancient P lineage), and disconnected from the surviving populations (ancient H lineage) along the Pacific coast of eastern Japan.

Oceanic currents are commonly the main hydrodynamic forces shaping population connectivity and phylogeographic structure of intertidal seaweeds (e.g., Alberto et al., 2011; Billot, Engel, Rousvoal, Kloareg, & Valero, 2003). The P lineages of *S. japonica* that likely originated from the northern refugium along the Japan Sea side of Hokkaido Island (*c*. 44°N) could expand to Sakhalin Island by the Tsushima Warm Current, and subsequently colonize the west coast of the Japan Sea by the Limen Cold Current after the LGM (Supporting Information Figure S1). When the Tsugaru Strait opened during the interglacial period, the Oyashio Cold Current branched into the Japan Sea and lowered the temperature and salinity (Oba et al., 1991; Wang, 1999). Given the cold tolerant of *S. japonica*, the ancient H lineage from the Pacific side of the Oshima Peninsula could likely traverse the Tsugaru Strait to the west coast of Hokkaido via the Oyashio Cold Current.

In addition to genetic divergence, the P and H lineages exhibited adaptive divergence in the morphology and physiology (Supporting Information Table S11). Morphologically, the kelp from Russia featured a slightly longer thallus (2.0–3.5 m) than the kelp from Japan (1.5–3.0 m) (Balakirev et al., 2012; Kawashima, 2012), which could be attributed to the 2–3 years life span of kelp from Russia compared with the 1–2 years life span of kelp from Japan.

### 4.2 Secondary contact in Sakhalin

Although an admixture‐like structure was detected based on the clustering method, it remains difficult to distinguish real admixtures (resulting from hybridization between divergent lineages) from shared ancestral polymorphisms (inherited from a common ancestor) without referring to a coalescent analysis (Tsuda, Nakao, Ide, & Tsumura, 2015). In this study, the ABC simulation favored the admixture model (Figure 2) and indicated that the admixture was generated by secondary contact between the groups H and P instead of being due to shared ancestral polymorphism from a common ancestor. This agrees with the coexistence of the two shared haplotypes H12 (mainly in group H) and H54 (mainly in group P) in the three Sakhalin populations (WB, WS, and SH) (Supporting Information Figures S3 and S4).

With reference to the kelp mitochondrial genetic structure, 51.13% of the molecular variance occurred among the four BAPS groups (Supporting Information Table S9). In contrast, the analyses based on microsatellite data revealed two genetic groups with admixed populations and only 16.5% of the nuclear variance occurred among groups, which is indicative of high levels of mitochondrial genetic structure in the absence of significant nuclear structure in *S. japonica*. Such “mito‐nuclear” discordance is common in secondary contact zones (Bonnet, Leblois, Rousset, & Crochet, 2017; Edgington, Ingram, & Taylor, 2016; Fontenot, Makowsky, & Chippindale, 2011; Toews & Brelsford, 2012). We suspect that this phenomenon could be caused by the different introgression degrees observed for the mitochondrial and nuclear markers (e.g., Neiva, Pearson, Valero, & Serrão, 2010). Hence, secondary contact in Sakhalin between the diverged H and P lineages might have facilitated this discordant introgression during the postglacial northward expansion. Another explanation is positive selection on the mitochondrial genome or adaptive mitochondrial introgression (e.g., Bonnet et al., 2017), which would be observed if introgressing populations (H lineages) are more fit than the populations (P lineage) adapted to the environment in Sakhalin.

### 4.3 Climate‐driven range shifts

Our niche model projections showed that mean sea surface temperature and chlorophyll concentration, which is positively related to water turbidity, are the most important environmental variable in discriminating suitable from nonsuitable habitat for the kelp *S*. *japonica*. Recent study has indicated that an increase in mean seawater temperature of 1–2°C could cause a marked decrease in the standing biomass of *S. japonica *(Gao, Endo, & Agatsuma, 2014). Temperature is the main factor that regulates seaweed growth and limits the geographic distribution of seaweed (Breeman, 1988; Harley et al., 2012). Light is another key limiting factor for the growth of coastal seaweed. Autotrophic biomass increases water turbidity and reduces light transmission. This might explain why the habitat suitability of *S. japonica *was negatively correlated with the concentrations of chlorophyll, as has been reported in at least two other seaweed species (Jueterbock et al., 2016; Méléder, Populus, Guillaumont, Perrot, & Mouquet, 2010).

Continued warming results in northward range shifts of seaweeds, especially of the cold‐temperate species (Assis, Serrao, Claro, Perrin, & Pearson, 2014; Neiva et al., 2014; Nicastro et al., 2013; Raybaud et al., 2013; Wernberg, Russell, Moore et al., 2011). After the LGM, rising sea levels opened the Tsushima Strait, which contributed to increases in sea temperature and salinity. As a result, the kelp populations expanded in the mid‐Holocene (~6 kya) far beyond the species’ current limit (Figure 3). This is not exceptional, as similarly vast range expansions in the mid‐Holocene were also described for the seaweed *Fucus vesiculosus* (Assis et al., 2014).

### 4.4 Implications for wild kelp conservation and germplasm utilization

*Saccharina japonica *is extensively used for food and industrial materials. It is mainly farmed commercially in China, Japan, and Korea. Domestication has reduced genetic diversity and narrowed the germplasm base of cultivated seaweeds (Huh, Lee, Lee, & Choi, 2004; Niwa & Aruga, 2006; Voisin, Engel, & Viard, 2005), and *S. japonica* is no exception (Zhang et al., 2017). Knowledge about the levels of diversity and evolutionary history of wild *S. japonica* populations can inform how and where to collect wild germplasm from the Japan Sea for cultivar selection and breeding in China, Korea, and Japan. For example, the kelp populations of high genetic diversity along the Hokkaido coast, south Honshu, and the Sakhalin coast (contact zone) are valuable resources for the long‐term management and breeding purposes of kelp aquaculture, and can be specifically collected and preserved ex situ.

The spatial population structure and location of climate refugia provide essential information for the conservation and management of kelp and the associated marine biodiversity. Ancient refugia are generally characterized by unique genetic variation and high genetic diversity, representing long‐term persistence (Assis, Lucas, Bárbara, & Serrao, 2016; Hewitt, 2004; Provan & Maggs, 2012). However, the ancient refugia of *S*. *japonica* along the south coast of the Japan Sea provided suitable habitat only for a short time period as the climate warmed (Figure 3). That the species’ range is retracting from these regions suggests that the populations are in a marginal environment and will progressively disappear because temperatures are becoming too warm. In the marine realm, both northward expansions (leading edge) and southern range contractions (trailing edge) represent common responses to future climatic oscillations (e.g., *F. ceranoides*, *Ecklonia cava,* *Pelvetia canaliculata*; Neiva et al., 2014; Neiva et al., 2012; Takao, Kumagai, Yamano, Fujii, & Yamanaka, 2015). The southern edge populations of *S*. *japonica* along the coast of the Tsugaru Strait between Hokkaido and the Honshu Peninsula are predicted to vanish by the year 2,100. Considering their high genetic diversity and higher extinction probability, low latitude genetic relics (populations at the southern edge of the range along Hokkaido and south Honshu) should be declared as valuable conservation units. Moreover, the kelp populations in these regions mainly include subspecies of *S. japonica *(*S. japonica *var *religiosa*), which are more resistant to high temperature (Kawashima, 2012). These heat‐resistant and early maturing kelp populations are potential germplasm resources with preservation value under the present global warming risk.

Hybridization has long been recognized as an important source of genetic variation that contributes to the evolution of novel phenotypes or adaptations to new environments (Arnold, Sapir, & Martin, 2008; Lewontin & Birch, 1966; Taylor, Larson, & Harrison, 2015). Hence, given their genetic admixture, the populations in Sakhalin should represent evolutionary significant units (ESU) with increased adaptability to new habitats opening up northwards along the coast of the Okhotsk Sea within the next 50–100 years (Figure 3).

## 5 CONCLUSION

The kelp *S. japonica* persisted in the south of the Japan Sea (*c*. 35–44°N) and along the Pacific coastlines of the Oshima Peninsula (Hokkaido) and Shimokita Peninsula (Honshu) during the LGM. The restriction of *S*. *japonica* to isolated refugia and the opening of the Tsushima Strait after the LGM likely played major roles in shaping the present distribution and phylogeographic diversification of this kelp. Most of the present range comprises the postglacial expansion zones, and the secondary contact zone between the two diverged lineages (H and P). Many populations of *S. japonica* are on the verge of local extinction due to climate‐induced increases in sea temperature. Further studies are needed to explore whether this seaweed has sufficient adaption or acclimation potential to mitigate the predicted climate change‐induced range shift or whether the species is at risk of losing its centers of genetic diversity.

## CONFLICT OF INTEREST

No authors have any conflict of interest to declare.

## Data Availability

DNA sequences have been deposited in the GenBank (accession numbers KT963093‐KT963144; MK227354‐MK227387). We include a large data package on Dryad Digital Repository (https://doi.org/10.5061/dryad.482f265) that contain the input files and result files for DIYABC, Maxent, and STRUCTURE, and the alignment file of mitochondrial data.
