# Supplementary material for: Phylogeographic diversification and postglacial range dynamics shed light on the conservation of the kelp Saccharina japonica
Source: Evol Appl. 2019 Jan 12;12(4):791–803. doi: 10.1111/eva.12756 (PMC6439492; doi:10.1111/eva.12756)
Supplement: Supplementary file 1 [file EVA-12-791-s001.pdf]

Phylogeographic diversification and postglacial range dynamics shed light on the conservation of the kelp *Saccharina japonica*

Jie Zhang, Jianting Yao, Zi-Min Hu, Alexander Jueterbock, Norishige Yotsukura, Tatiana N Krupnova, Chikako Nagasato, Delin Duan

Table of Contents:

**Figure S1**	Page 2
**Figure S2**	Page 3
**Figure S3**	Page 4
**Figure S4**	Page 5
**Figure S5**	Page 6
**Figure S6**	Page 7
**Figure S7**	Page 8, 9
**Figure S8**	Page 10,11
**Figure S9**	Page 12,13
**Figure S10**	Page 14
**Figure S11**	Page 15
**Figure S12**	Page 16
**Table S1**	Page 17
**Table S2**	Page 18
**Table S3**	Page 19
**Table S4**	Page 20, 21
**Table S5**	Page 22, 23
**Table S6**	Page 24
**Table S7**	Page 25
**Table S8**	Page 26
**Table S9**	Page 27
**Table S10**	Page 28
**Table S11**	Page 29

Figure S1 The distribution of wild *Saccharina japonica*. The pink line indicates the warm Current and the blue line shows the cold Current.

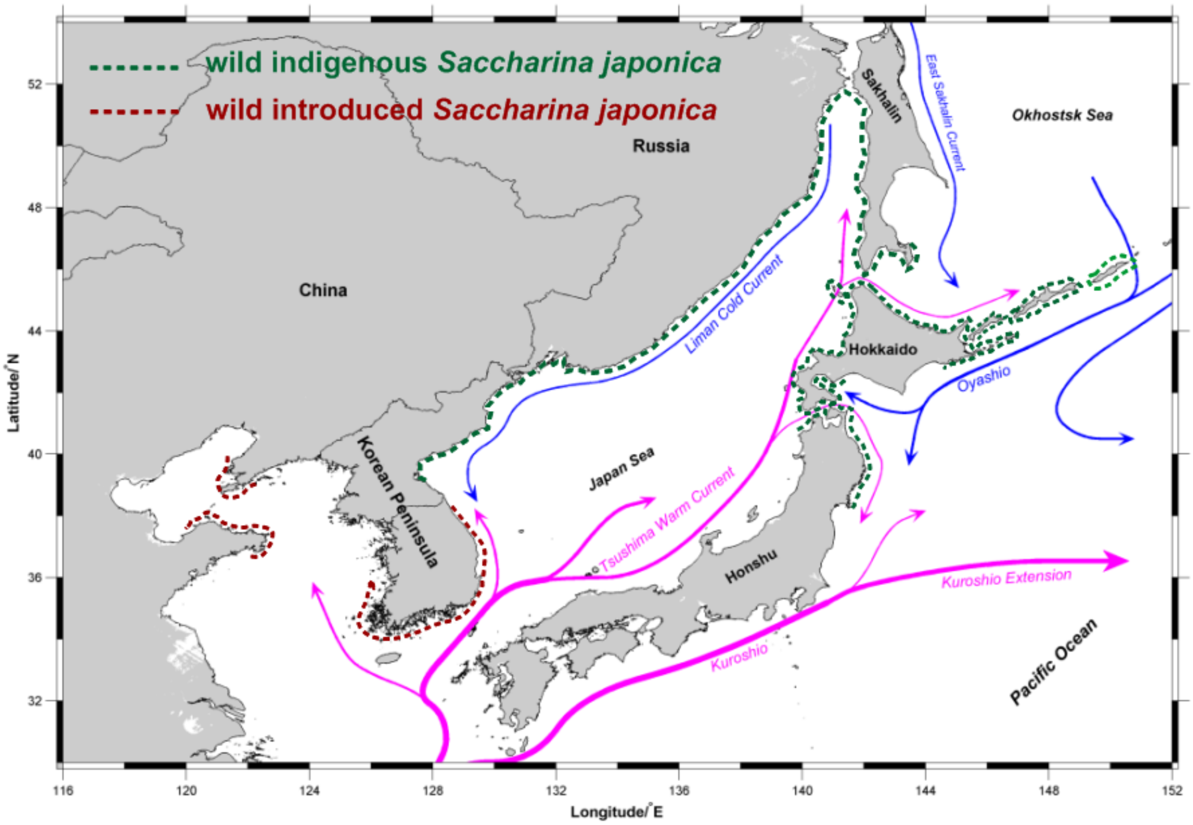

Figure S2 Coastal background (pseudo-absence) sites providing information on environmental conditions within the distributional range of *Saccharina japonica*.

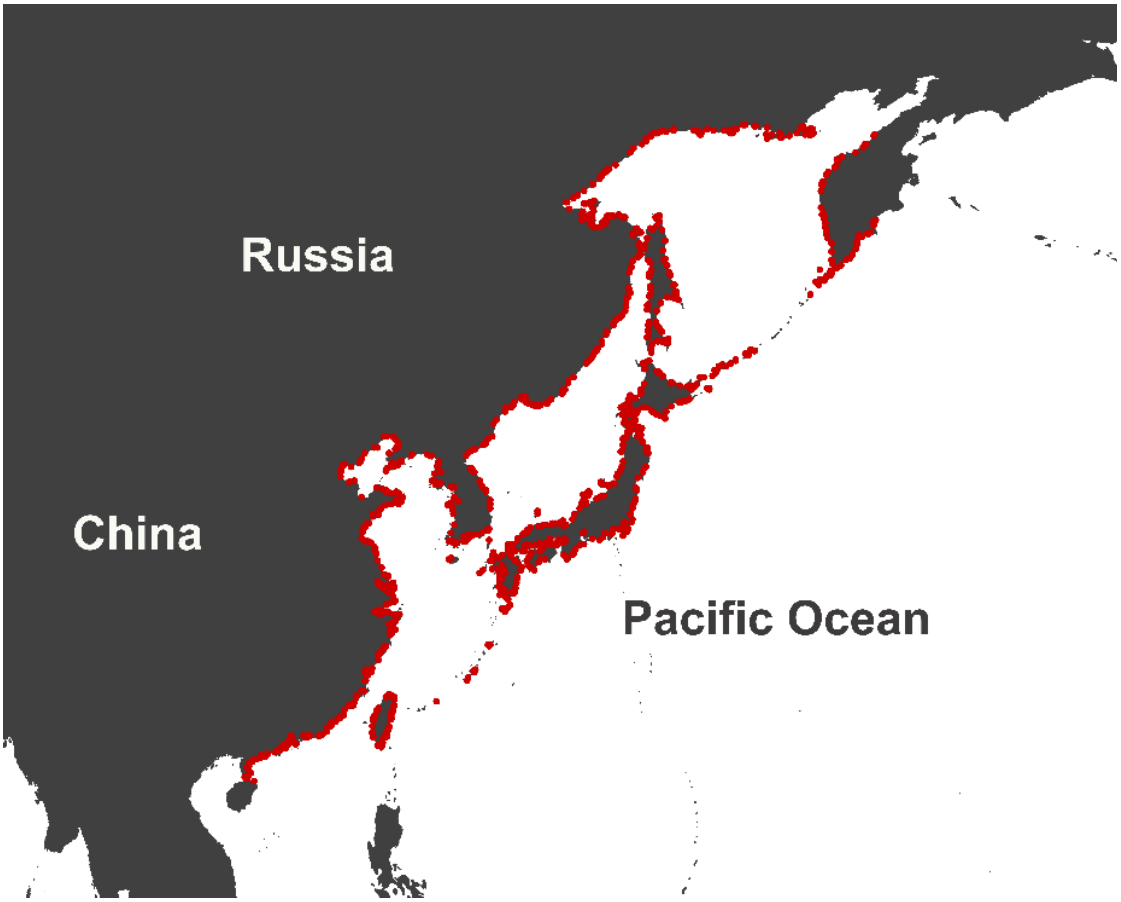

Figure S3 Geographic distribution of mtDNA haplotypes in *Saccharina japonica*.

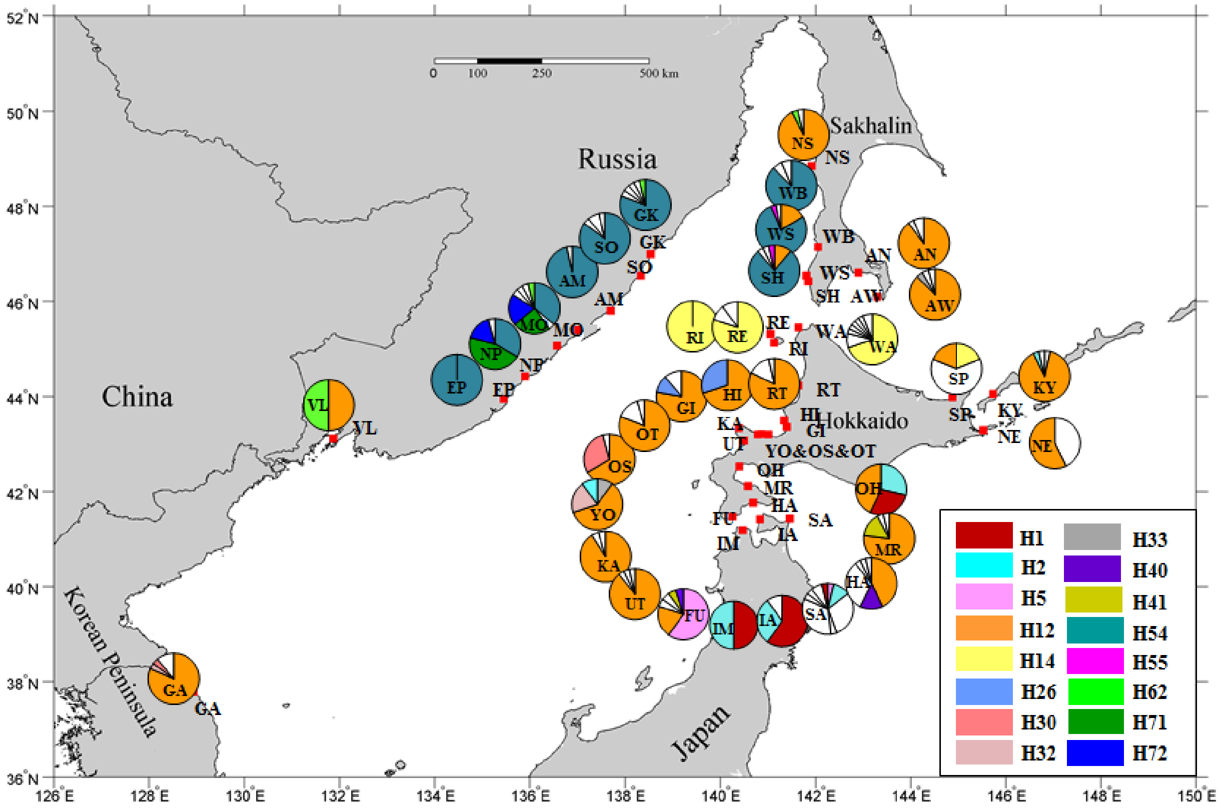

Figure S4 The median-joining network constructed from mtDNA haplotypes. Each connecting line indicates one mutation step between haplotypes; Missed or unsampled haplotypes are represented by small solid black squares.

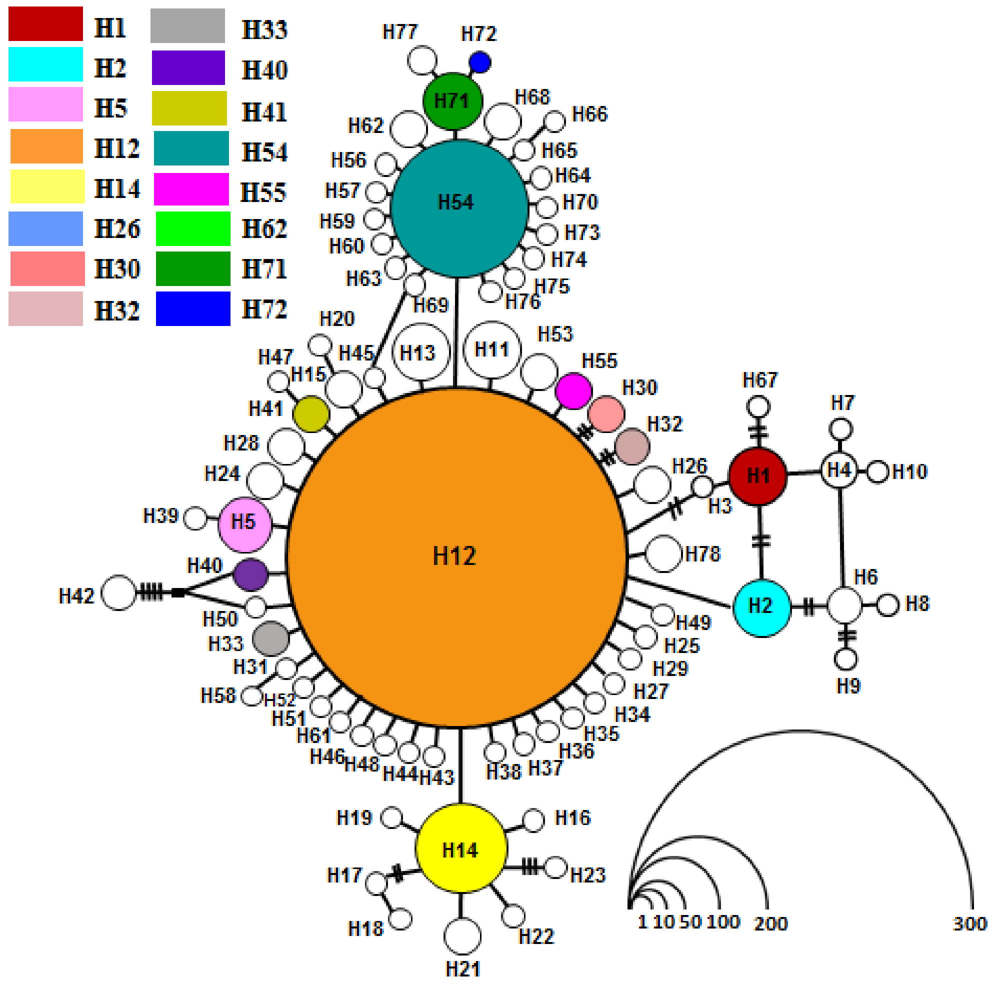

Figure S5 Determination of the optimal K values for *Saccharina japonica* populations implemented in STRUCTURE HARVESTER (http://taylor0.biology.ucla.edu/structureHarvester/) using the ΔK method.

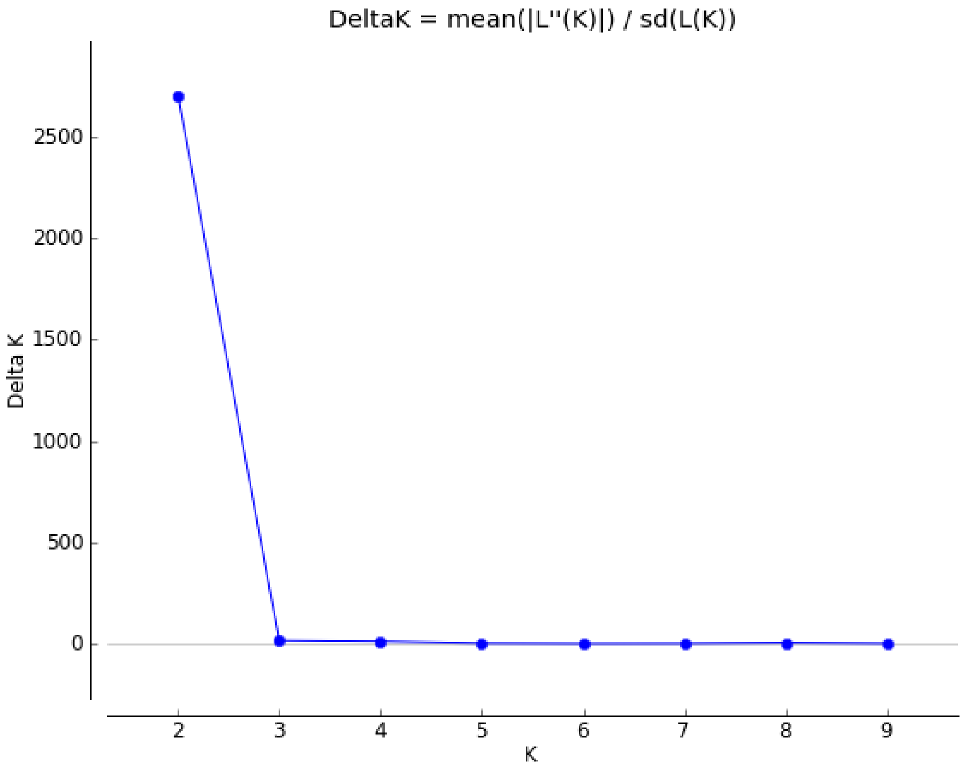

Figure S6 Genetic structure based on BAPS (K=4) and STUCTURE (K=2) analysis

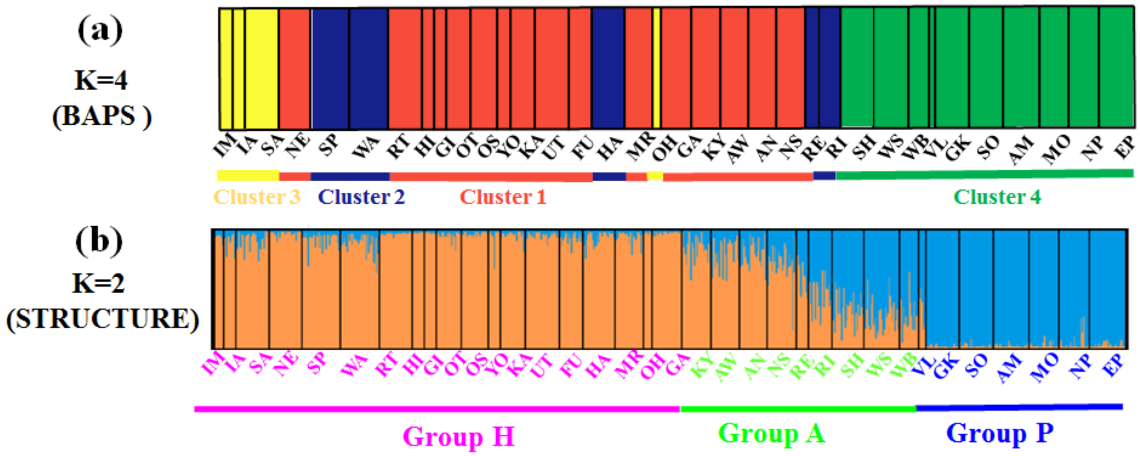

Figure S7 DIYABC analyses based on combined data for stage 1. (a) principle component analysis of summary statistics for scenario 1-5 prior sets; (b) principal component analysis of test parameters when processing model checking for the most likely scenario 5; (c) the Posterior Probability (y-axis) of estimates of the parameters in scenario 5.

(a)

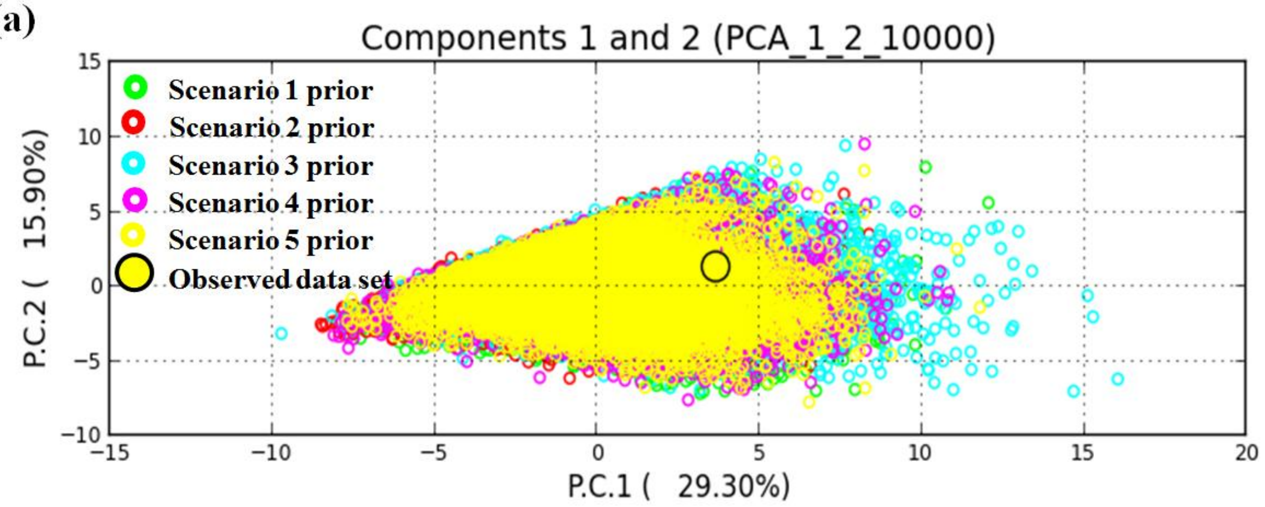

(b)

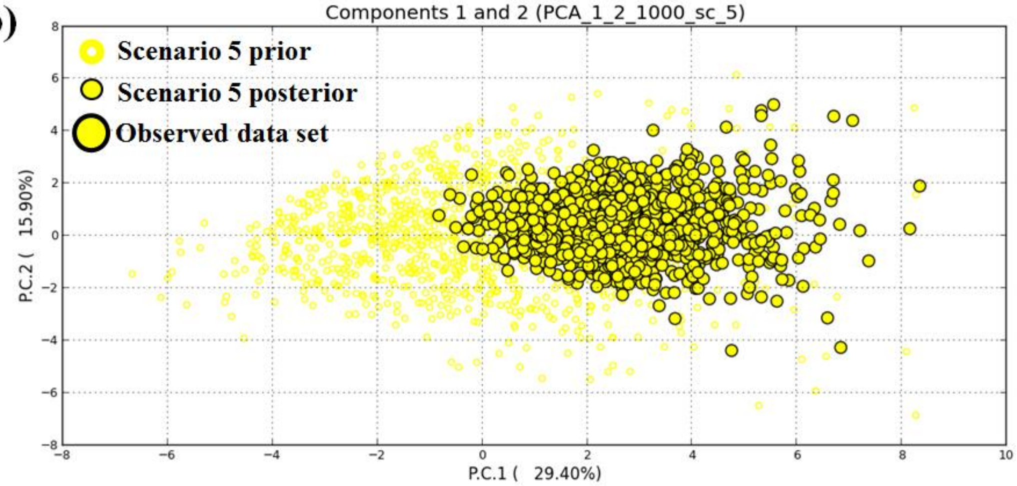

(c)

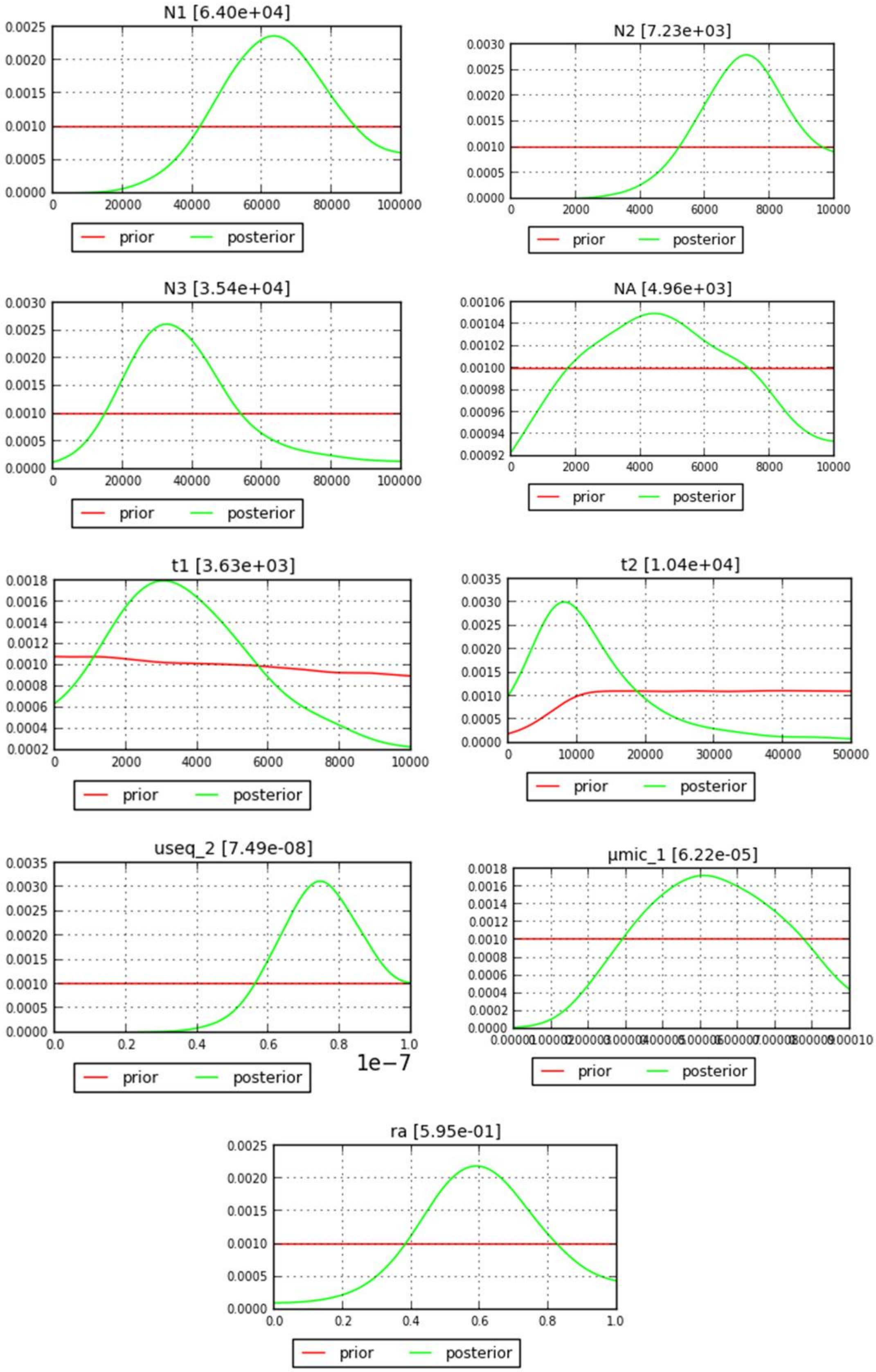
N1 [6.40e+04]
prior
posterior
N2 [7.23e+03]
prior
posterior
N3 [3.54e+04]
prior
posterior
NA [4.96e+03]
prior
posterior
t1 [3.63e+03]
prior
posterior
t2 [1.04e+04]
prior
posterior
useq_2 [7.49e-08]
1e−7
prior
posterior
µmic_1 [6.22e-05]
prior
posterior
ra [5.95e-01]
prior
posterior

Figure S8 DIYABC analyses based on combined data for stage 2. (a) principle component analysis of summary statistics for scenario 6-7 prior sets; (b) principal component analysis (PCA) of test parameters when processing model checking for the most likely scenario 6; (c) the Posterior Probability (y-axis) of estimates of the parameters in scenario 6.

(a)

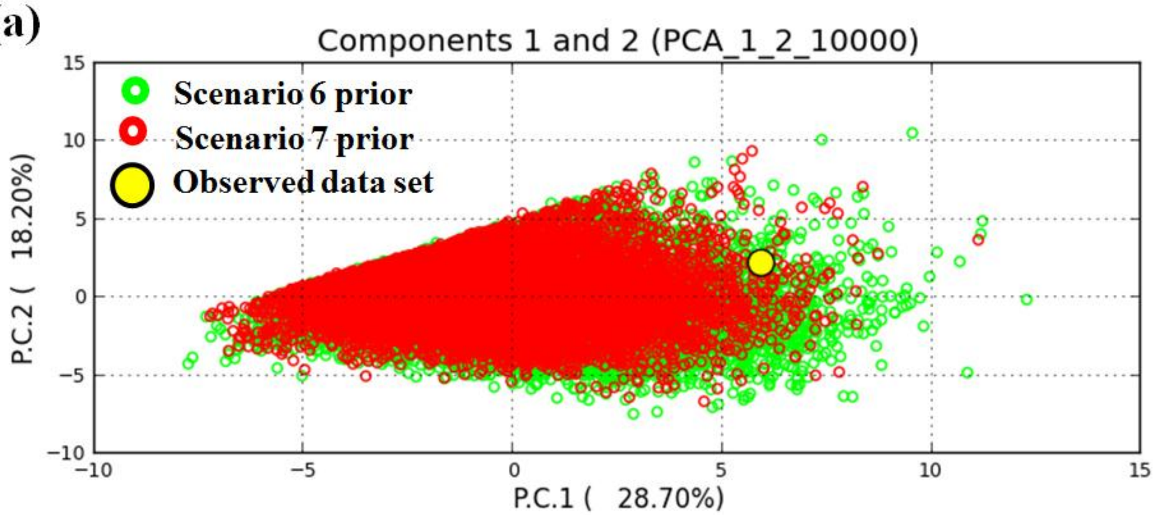

(b)

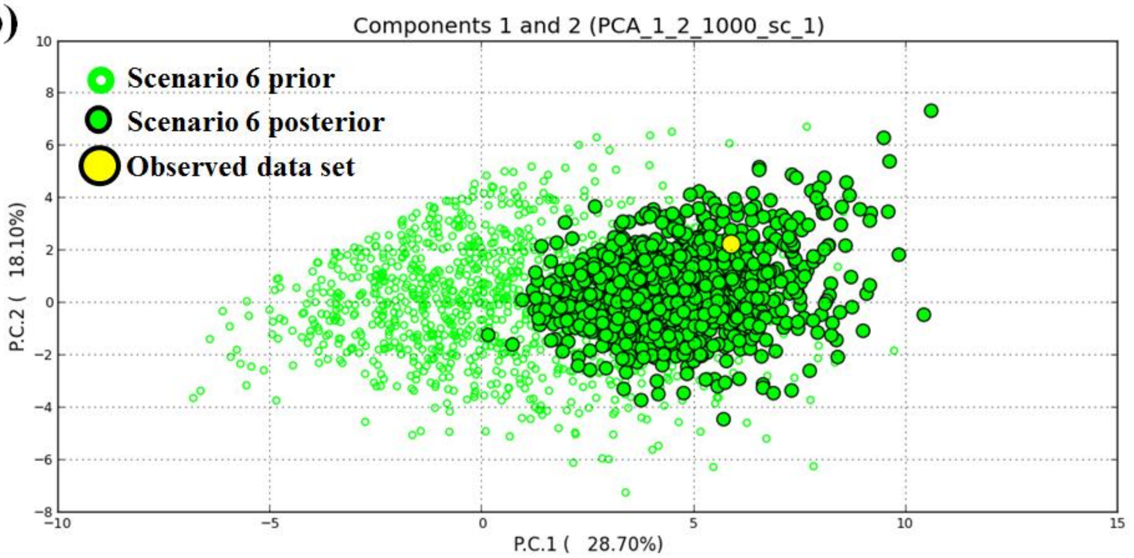

(c)

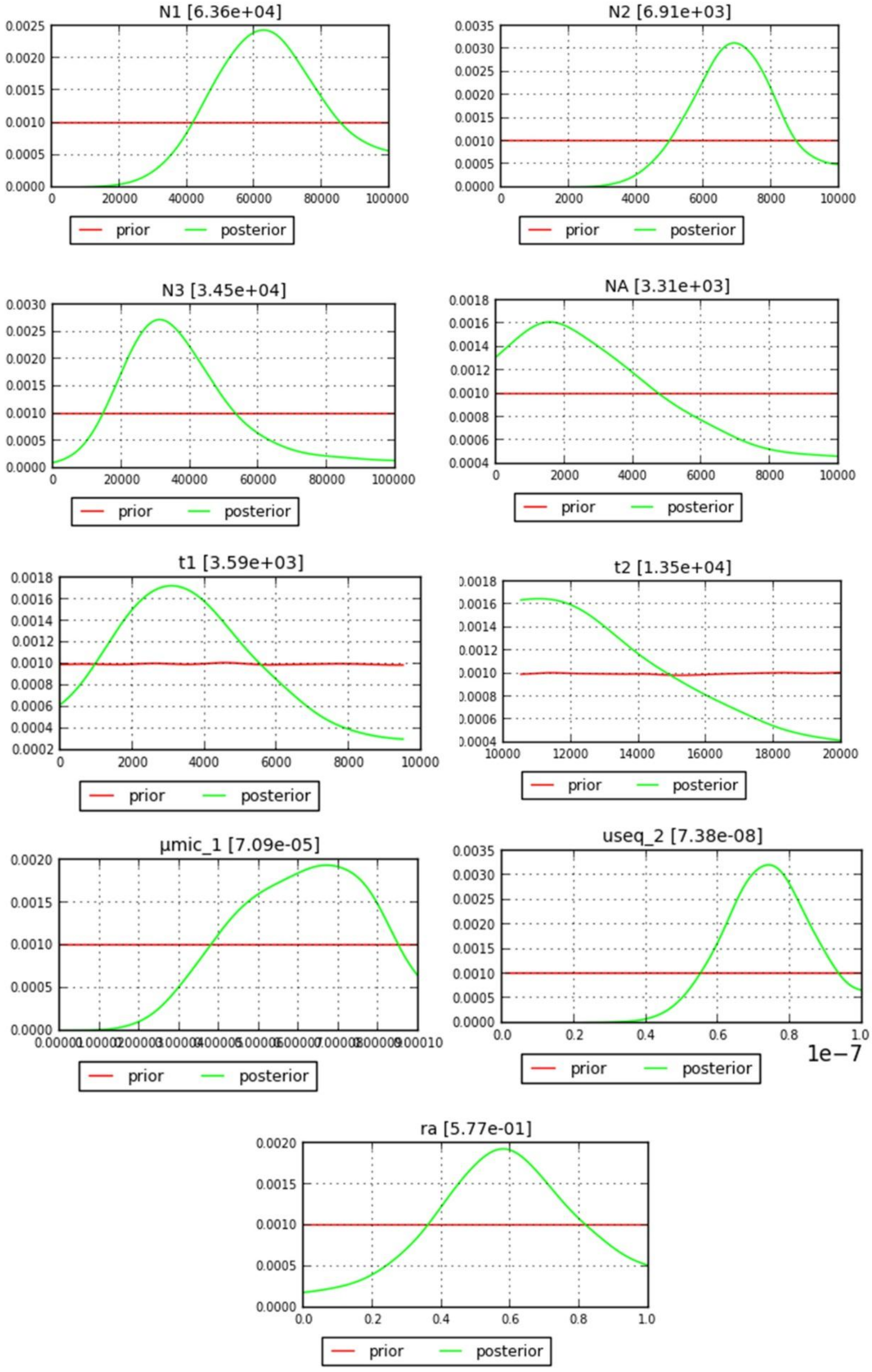

N1 [6.36e+04]
prior
posterior
N2 [6.91e+03]
prior
posterior
N3 [3.45e+04]
prior
posterior
NA [3.31e+03]
prior
posterior
t1 [3.59e+03]
prior
posterior
t2 [1.35e+04]
prior
posterior
μmic_1 [7.09e-05]
prior
posterior
useq_2 [7.38e-08]
1e−7
prior
posterior
ra [5.77e-01]
prior
posterior

Figure S9 DIYABC analyses based on combined data (SSR and mitochondrial DNA) and SSR. (a) logistic regression plot showing the Posterior Probability of each scenario (model 1-5) based on combined data; (b) logistic regression plot showing the Posterior Probability of each scenario (model 1-5) based on SSR data; (c) logistic regression plot showing the Posterior Probability of each scenario (model 6-7) based on combined data; (d) logistic regression plot showing the Posterior Probability of each scenario (model 6-7) based on SSR data.

(a)

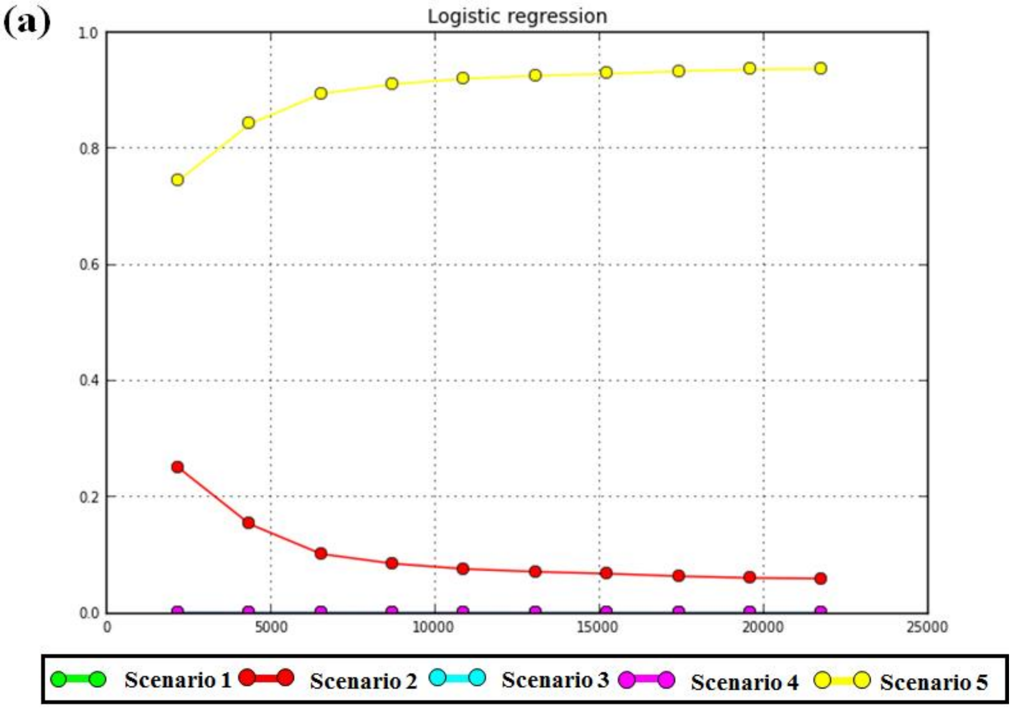

(b)

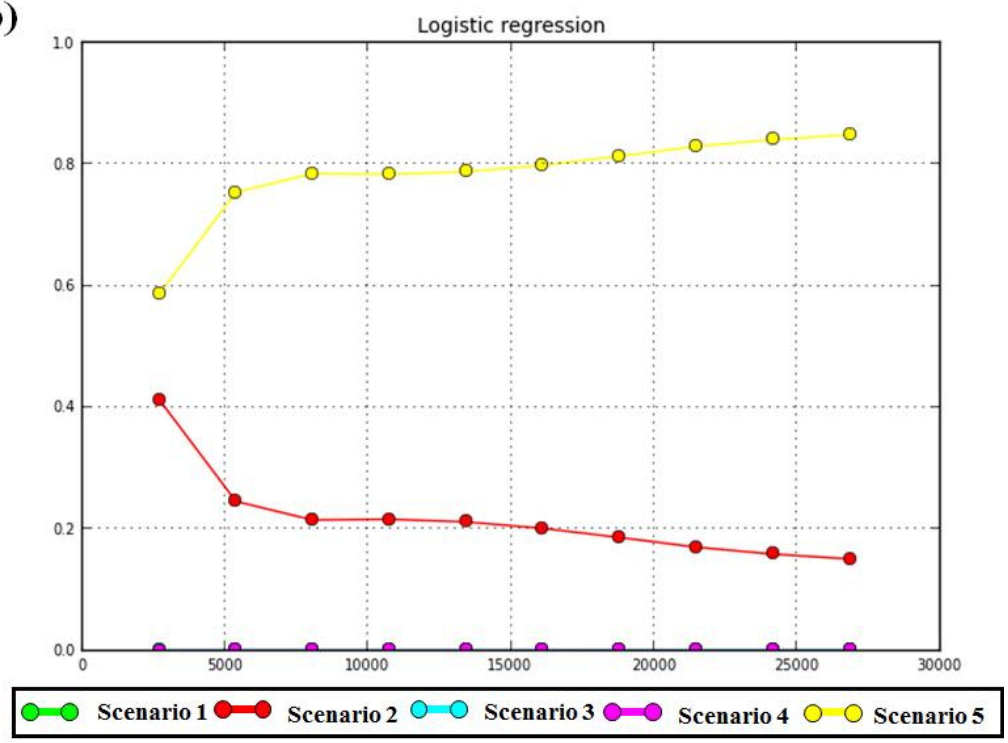

(c)

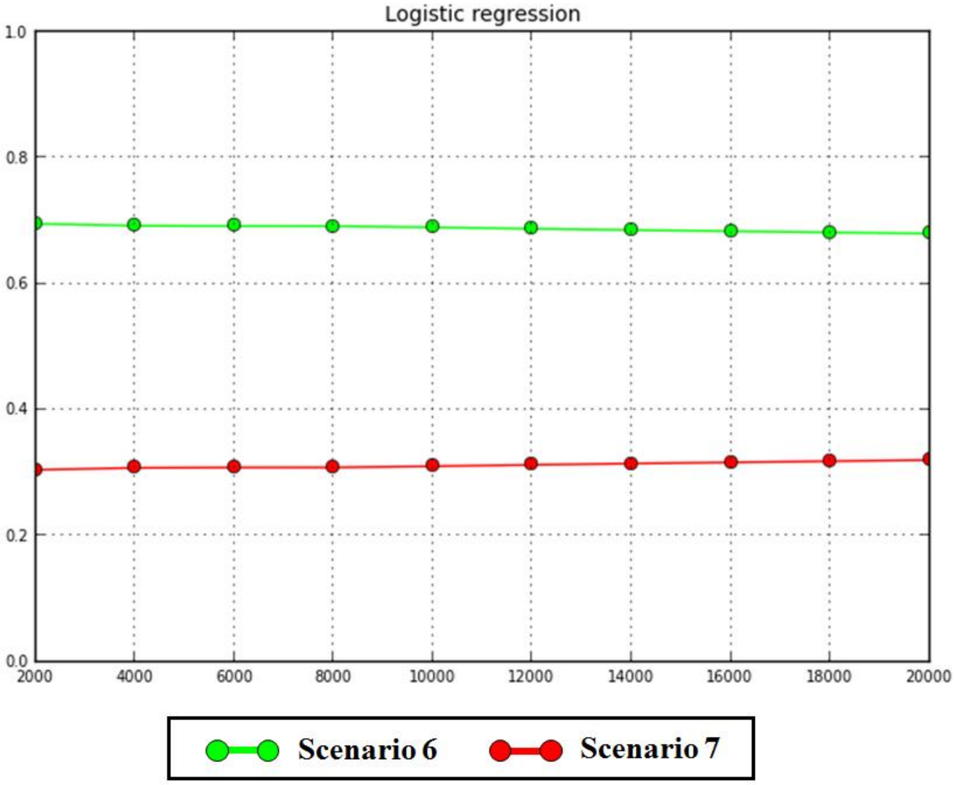
Logistic regression
1.0
0.8
0.6
0.4
0.2
0.0
2000
4000
6000
8000
10000
12000
14000
16000
18000
20000
Scenario 6
Scenario 7

(d)

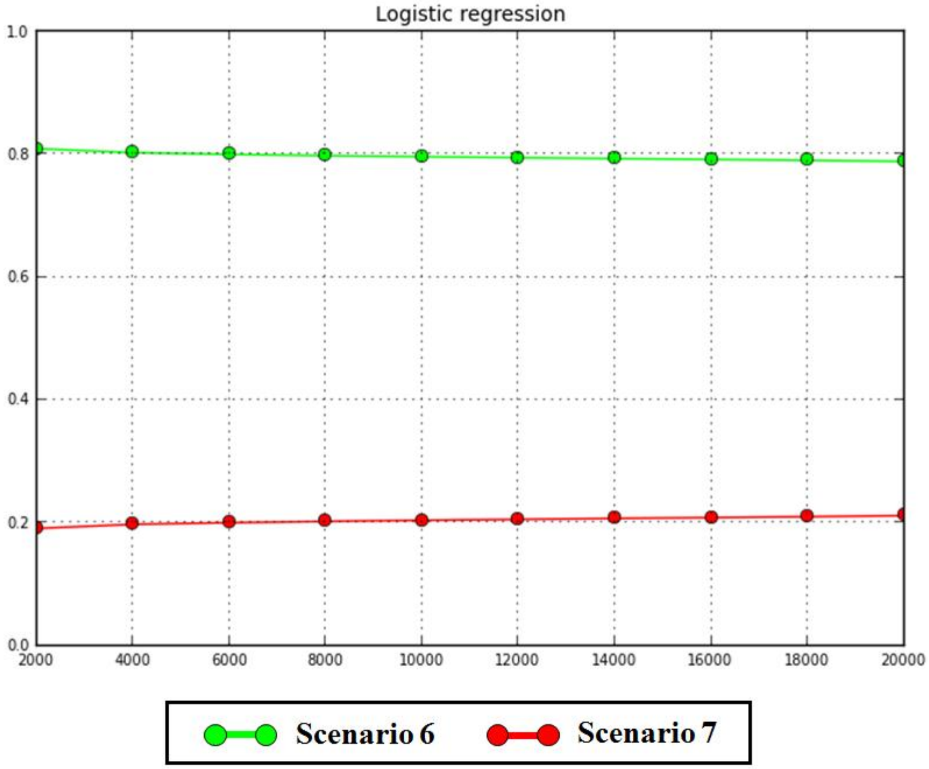
Logistic regression
1.0
0.8
0.6
0.4
0.2
0.0
2000
4000
6000
8000
10000
12000
14000
16000
18000
20000
Scenario 6
Scenario 7

Figure S10 Response curves of the three environmental variables that were chosen for niche modelling. The curves show how the mean (in red) logistic probability of presence of *Saccharina japonica* depends on each of the three variables over ten replicate models; the range of two standard deviations are represented as a blue shade. The scale for each of the three variables is set by the range of values sampled by the background points in our study area. SST: Sea Surface Temperature"

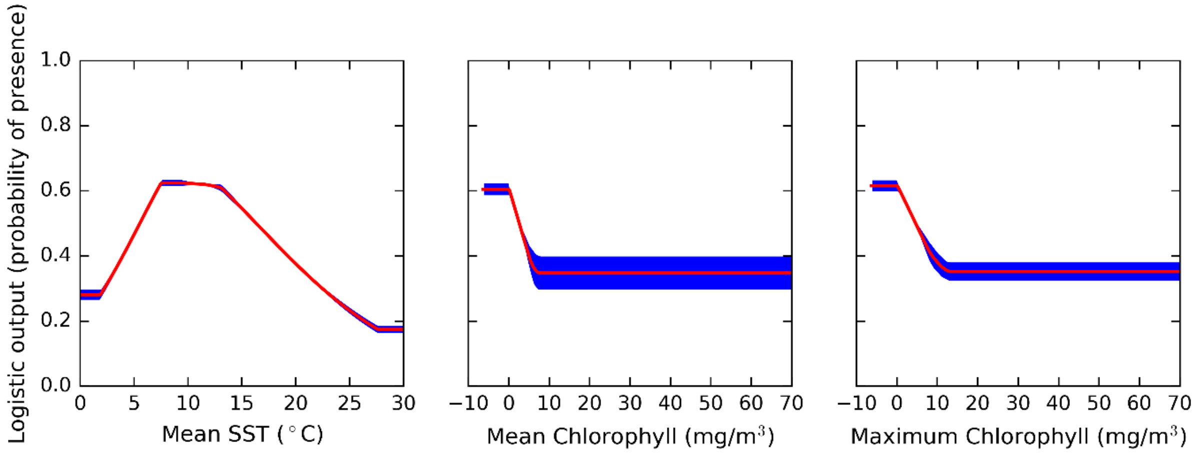

Figure S11 Comparison of the performance of niche models that differed the beta-multiplier (1 to 10 with increments of 0.5). Model-performance was estimated by the area under the receiver operating characteristic estimated from test data (AUC.Test) (Fielding & Bell, 1997). Each model with a certain beta-multiplier started with 20 variables and was simplified in a stepwise fashion by removing those variables with low contribution-scores (<5%) and high correlation with other variables (correlation coefficients >0.9 or <-0.9). The number of variables in each model is encoded both by color and size. The model of highest performance (based on AICc (Akaike, 1974), not AUC.Test) is marked in red.

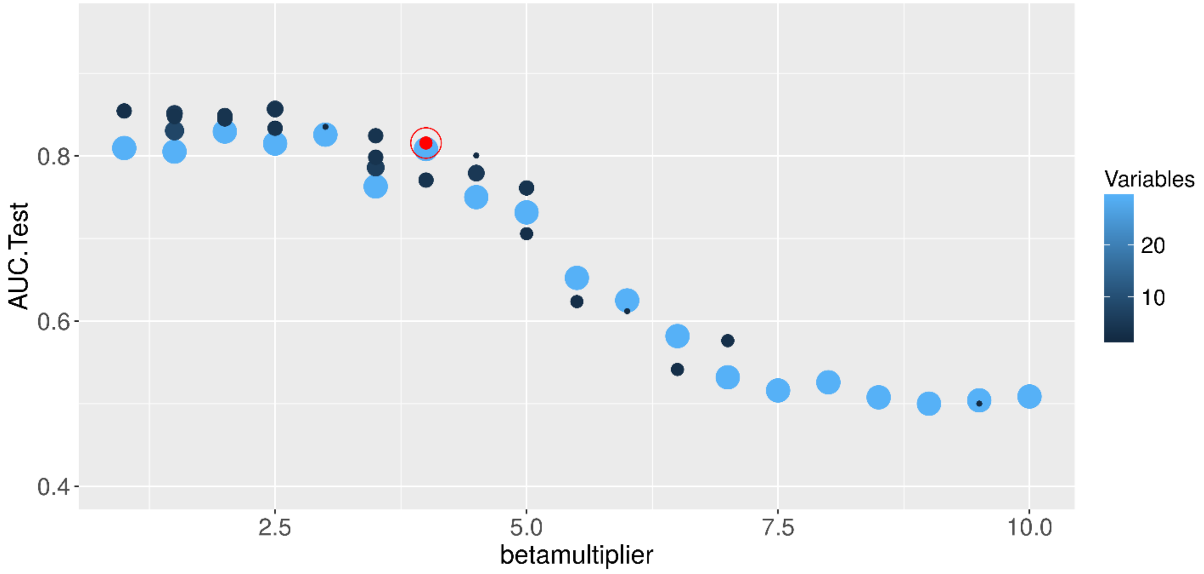

Figure S12 Comparison of the performance of niche models that differed in the beta-multiplier (1 to 10 with increments of 0.5). Model-performance was estimated by the sample-size-adjusted Akaike information criterion (AICc) (Akaike, 1974). AICc values could not be calculated where the number of model parameters exceeded the number of occurrence sites. Each model with a certain beta multiplier started with 20 variables and was simplified in a stepwise fashion by removing those variables with low contribution-scores (<5%) and high correlation with other variables (correlation coefficients >0.9 or <-0.9). The number of variables in each model is encoded both by color and size. The model of highest performance (lowest AICc) is marked in red.

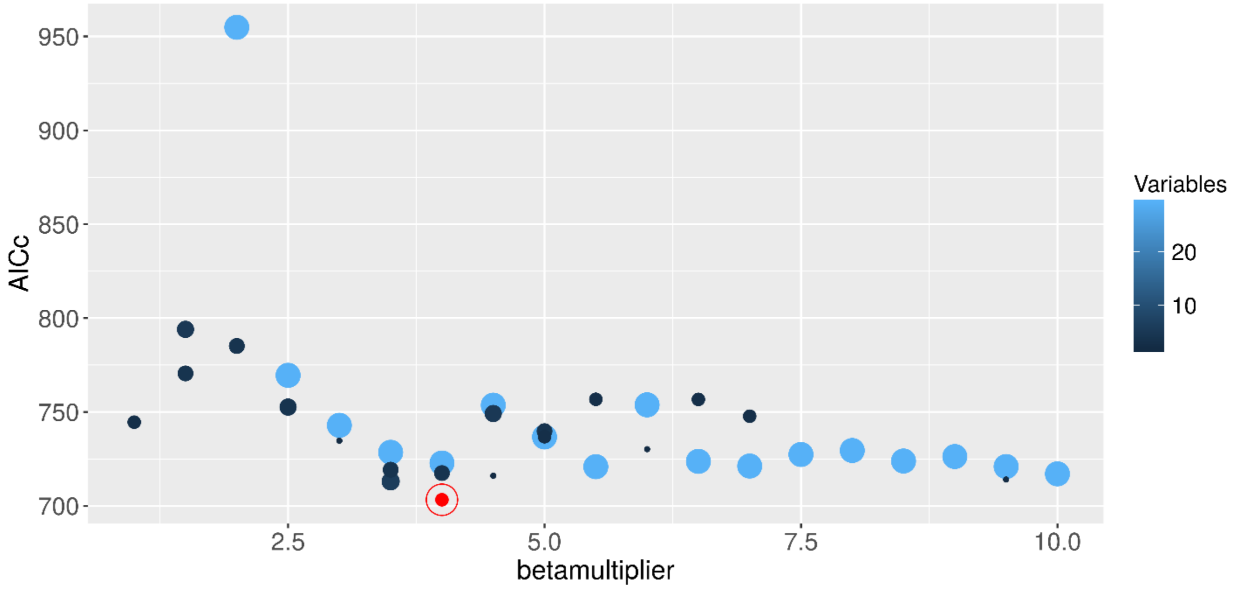

Table S1 Sampling locations of 35 *Saccharina japonica* populations.

Code	Number	Collection site	Latitude and longitude	Data
IM	8	Imabetsu, Aomori pref.	N41°10′57″ E140°28′35″	2014.11.06
IA	10	Ishoya, Aomori pref.	N41°24′40″ E140°50′13″	2014.11.06
SA	28	Shiriya, Aomori pref.	N41°25′50″ E141°27′37″	2014.11.04
NE	28	Nemuro, Hokkaido	N43°17′38″ E145°31′24″	2012.07.02
SP	33	Shiretoko Peninsula, Hokkaido	N43°58′41″ E144°52′32″	2012.06.30
WA	34	Wakkanai, Hokkaido	N45°27′01″ E141°38′42″	2012.12.06
RE	10	Rebun, Hokkaido	N45°18′47″E141°03′20″	2014.11.06
RI	20	Rishirifuji, Hokkaido	N45°12′02″E141°08′03″	2014.11.06
RT	30	Rikibiru, Tomamae, Hokkaido	N44°13′54″E141°38′35″	2014.11.06
HI	10	Hamamasu, Ishikari, Hokkaido	N43°30′07″E141°20′46″	2014.11.06
GI	10	Gokibiru, Ishikari, Hokkaido	N43°20′09″E141°25′00″	2014.11.06
OT	21	Otaru, Hokkaido	N43°12′00″E141°00′47″	2012.07
OS	25	Oshoro, Hokkaido	N43°12′24″E140°51′30″	2012.07
YO	10	Yoichi, Hokkaido	N43°11′54″ E140°47′32″	2012.07.17
KA	22	Kamui cape, Hokkaido	N43°20′17″E140°23′27″	2012.07.17
UT	30	Usubetsu, Tomari, Hokkaido	N43°04′20″ E140°29′48″	2014.11.26
FU	20	Fukushima, Hokkaido	N41°28′23″E140°15′33″	2014.11.06
HA	29	Hakodate, Hokkaido	N41°46′00″E 140°41′25″	2011.10
MR	26	Mori, Hokkaido	N42°06′59″E 140°35′07″	2011.10
OH	10	Oshamambe, Hokkaido	N42°31′40″E140°23′49″	2012.07
KY	30	Kyrilian island	N44°03′18″E145°43′48″	2011.07
AW	24	Aniva Bay (west), Sakalin	N46°06′00″ E143°18′00″	2011.09
AN	30	Aniva Bay (north), Sakalin	N46°36′00″ E142°54′00″	2011.09
SH	40	Shebunino, Aniva Bay, Sakalin	N46°25′03″E141°51′01″	2013.9.10
WS	31	Western Sakalin	N46°32′29″E141°48′40″	2011.09
WB	16	Western bay of Sakalin	N47°08′29″E142°03′31″	2011.09
NS	26	Northern Sakalin	N48°50′35″E141°55′12″	2011.09
GK	28	Gilak cape, Primorsky	N46º59′00″ E138º33′00″	2014.12
SO	30	Sosunova cape, Primorsky	N46º32′00″ E138º20′00″	2014.12
AM	30	Amgu, Primorsky	N45º49′00″ E137º41′00″	2014.12
MO	30	Mosolova cape, Primorsky	N45º05′00″ E136º34′00″	2014.12
NP	31	Northern Primorsky	N44°25′00″ E135°54′00″	2014.10
EP	33	Eastern Primorsky	N43°57′00″ E135°27′00″	2012.10
VL	7	Vladivostok, Primorsky	N43°06′54″E131°52′12″	2011.09
GA	30	Gangneung, Korea	N37°47′24″E128°55′48″	2013.06

Table S2 The primer information and genetic indices of 24 SSR markers.

SSR	Motif	Forward primer (5'-3')	Reverse primer (5'-3')	*N*	A_R	*PIC*	H_O	H_E
SJ3	(CTG)10	CTTATTCGTGCTGCGTTCATAC	AGTTTGAGCCTCTGCGTCAT	19	3.53	0.52	0.42	0.41
SJ13	(GTCGG)7	CAGCAGATGGCGACTTACTTTAG	GTCTTGACGGAGCGTTTGAT	9	2.87	0.34	0.24	0.29
SJ80	(GCA)10	CTCCGACTGCCATACTCTTTGAC	CTTCGTGCTTACGTATATCCGAGTT	24	7.24	0.88	0.74	0.70
SJ20	(TTGCT)7	AGAGAACGCGGAGTGGAACT	CGCACTCGTCACAACGTGTAT	20	4.53	0.66	0.56	0.53
SJ66	(CCATA)5	GACTCCTCCGACTCGATCCT	ATAAGTTCTCCCGAAGCTGTTTCT	14	3.61	0.57	0.42	0.45
SJ95	(TCGG)5	CGGTTAGGCACTCCATTGTATC	GCCAGTCGAAACTCAATACTCATAC	35	7.19	0.84	0.67	0.70
SJ133	(CAG)8	AGCGATAATCAGGAAATCGTCTC	GTTTGGGTTGTTCTGTCACATTC	14	3.27	0.55	0.40	0.41
SJ106	(TGT)7	ACAAGAAGAGCGCACAGAGAAT	ATACGTACGTGATAGCTGGACTTC	11	2.79	0.35	0.29	0.32
SJ132	(TAG)11	GGTATACTTTCTCCGCCATCAAG	GCACCGAGTTTCTACACCTGTT	45	9.01	0.94	0.91	0.89
SJ52	(AGGC)5	ATGAAGGCAATCATCATCAGGT	TAGCACCAACGCTCTAGAATAGAAG	10	4.36	0.68	0.49	0.51
SJ31	(CAG)6	AAGCAGCAGCCATCATGTTAC	CTGGTCAAGTCTCTGCTCTCATC	9	2.61	0.36	0.24	0.25
SJ93	(AAAC)7	CACCCTTATCATCCCTGTTCAAG	TTCGGGACAAGAGTGATACATAGTT	6	3.34	0.60	0.36	0.38
SJ86	(TTAT)6	AGTAGAAGCGGCAGTAGGTAGGAC	AAGAGCAACACATACACATGCATAC	15	4.66	0.71	0.48	0.51
SJ102	(TGC)6	TACTGTACTGCAGTGCTTGCGATTG	CAGCAGCACTACTCCTAGGTACATT	16	5.20	0.70	0.47	0.52
SJ99	(ACAG)5	AAAGGTTGGACAGACACGATATG	ATCCCTTTCTCTTTCTCGTTCTCT	27	3.22	0.40	0.25	0.31
SJ110	(GTA)7	GGACGAAGTTATGGACTCGTTTC	GTATCACCCGTAGTTTCCTCACC	23	6.33	0.83	0.70	0.70
SJ167	(GCT)6	AAGGAGAGGAGCAGTGTGTGTT	GATGTGCGAACGCTGAGAC	8	2.89	0.44	0.29	0.35
SJ101	(CGTA)8	ACAACGCTCCCAAGAGACTG	GGTCGCATGGTAAGCTCTTCT	12	4.78	0.73	0.49	0.51
SJ21	(CCTTC)7	TTTGTCCGCTCTCGCTCTT	GATGTCCTTGCCCTTGAAATAC	16	5.14	0.75	0.51	0.60
SJ113	(TGC)9	GAACGAACGGACGAATCGTAT	GTGGACCACAGGGAGAGAGAT	17	5.05	0.72	0.39	0.48
SJ125	(GAAG)7	CCTCAGCTTCTCGATCCTCTT	CTTTGGTTGTCGTTGTTGTTATCC	8	3.03	0.43	0.26	0.29
SJ24	(GAACC)7	CACGTCTGCTTCTGCTCTACAAC	CGTAGATGATGAACAGGCTGAAC	21	6.57	0.85	0.70	0.73
SJ114	(CAC)9	AGCAGCAGAGCTGGAGACTG	GTCGTGTCCGAGTAGGAGGTTAG	11	4.02	0.66	0.32	0.34
SJ136	(TGTCC)7	GGGTATCTCTCGGAATGATAGAAC	AGAGCTAGCAGTAGCTGTGGAAAT	59	8.81	0.92	0.54	0.76

N, number of alleles; A_R, allelic richness (allelic richness per locus and population based on minimum sample size of 6 diploid individuals); *PIC*, polymorphism information content; H_O, observed heterozygosity; H_E, expected heterozygosity.

Table S3 Prior distributions of parameters for each scenario in stage1 and stage 2 based on SSR data and combined data (mitochondrial DNA and SSR).

Parameter	Prior distribution
**Stage 1 (model 1-model 5)**	
N1: population size in the Hokkaido and Honshu (H)	U(10, 100000)
N2: population size in Primorsky of Far-eastern Russia (P)	U(10, 10000)
N3: admixture population size in Sakhalin of Far-eastern Russia (A)	U(10, 100000)
NA: population size for ancestral populations	U(10, 10000)
t_2: time of divergence	U(10, 50000)
t_1: time of admixture	U(10, 10000)
μ_1: mean mutation rate per generation for SSR	U(10^{-5}, 10^{-4})
μ_2: mean mutation rate per generation for mitochondrial DNA	U(10^{-9}, 10^{-7})
ra: admixture rate	U(0.001, 0.999)
**Stage 2 (model6-model7)**	
N1: population size in the Hokkaido and Honshu (H)	U(10, 100000)
N2: population size in Primorsky of Far-eastern Russia (P)	U(10, 10000)
N3: admixture population size in Sakhalin of Far-eastern Russia (A)	U(10, 100000)
NA: population size for ancestral populations	U(10, 10000)
t_3: time of post-LGM divergence	U(10, 9500)
t_2: time of pre-LGM divergence	U(10500, 20000)
t_1: time of populations admixture	U(10, 9500)
ra: admixture rate	U(0.001, 0.999)
μ_1: mean mutation rate per generation for SSR	U(10^{-5}, 10^{-4})
μ_2: mean mutation rate per generation for mitochondrial DNA	U(10^{-9}, 10^{-7})

U, uniform distribution;

Table S4 Occurrence records of *Saccharina japonica*

NO	Location	Country	longitude	latitude	Reference
1	Mori, Hokkaido	Japan	140.585	42.117	Zhang et al., 2015
2	Hakodate, Hokkaido	Japan	140.69	46.767	Zhang et al., 2015
3	Nemuro, Hokkaido	Japan	145.523	43.294	Zhang et al., 2015
4	Kushiro, Hokkaido	Japan	144.12	42.963	Sample Collection
5	Shiretoko Peninsula, Hokkaido	Japan	144.876	43.978	Zhang et al., 2015
6	Oshamambe, Hokkaido	Japan	140.397	42.528	Zhang et al., 2015
7	Yoichi, Hokkaido	Japan	140.792	43.198	Zhang et al., 2015
8	Kamui cape, Hokkaido	Japan	140.391	43.338	Zhang et al., 2015
9	Otaru, Hokkaido	Japan	141.013	43.2	Zhang et al., 2015
10	Oshoro, Hokkaido	Japan	140.858	43.201	Zhang et al., 2015
11	Wakkanai, Hokkaido	Japan	141.645	45.45	Zhang et al., 2015
12	Wakkanai, Hokkaido	Japan	141.679	45.411	Zhang et al., 2015
13	Shiogama, Fukushima	Japan	141.271	41.484	Yotsukura et al., 2016
14	Himukai(Hyuga), Fukushima	Japan	140.259	41.473	Yotsukura et al., 2016
15	Ishoya, Aomori prefecture	Japan	140.837	41.411	Yotsukura et al., 2016
16	Imabetsu, Aomori prefecture	Japan	140.476	41.183	Yotsukura et al., 2016
17	Gokibiru, Ishikari	Japan	141.417	43.336	Yotsukura et al., 2016
18	Hamamasu, Ishikari	Japan	141.346	43.502	Yotsukura et al., 2016
19	Kutukata, Rishirifuji	Japan	141.135	45.129	Yotsukura et al., 2016
20	Shinminato, Rishirifuji	Japan	141.134	45.2	Yotsukura et al., 2016
21	Kafuka, Rebun	Japan	141.134	45.313	Yotsukura et al., 2016
22	Rikibiru, Tomamae	Japan	141.643	44.232	Yotsukura et al., 2016
23	Oohama, Iwanai	Japan	140.491	42.961	Yotsukura et al., 2016
24	Rikibiru, Tomamae	Japan	141.643	44.232	Yotsukura et al., 2016
25	Shiriya, Aomori prefecture	Japan	141.46	41.431	Yotsukura et al., 2016
26	Usubetsu, Tomari	Japan	140.497	43.072	Yotsukura et al., 2016
27	Akkeshi, Hokkaido	Japan	144.85	43.05	Yotsukura et al., 2010
28	Funairijima, Miyagi prefecture	Japan	141.501	38.23	Gao et al., 2016
29	Gangneung South Korea	South Korea	128.93	37.79	Zhang et al., 2015
30	Wonsan, North Korea	North Korea	127.527	39.433	Tseng, et al., 1962
31	Weihai city, Shandong	China	122.59	37.165	Zhang et al., 2015
32	Yantai city, Shandong	China	121.567	37.49	Zhang et al., 2015
33	Zhangzidao, Liaoning	China	122.717	39.02	Zhang et al., 2015
34	Qingdao, Shandong	China	120.32	36.07	Bi et al., 2011
35	Kyrilian island	Russia	145.73	44.055	Zhang et al., 2015
36	Aniva Bay (west), Sakalin	Russia	143.3	46.1	Zhang et al., 2015
37	Aniva Bay (north), Sakalin	Russia	142.9	46.6	Zhang et al., 2015
38	Shebunino, Aniva Bay, Sakalin	Russia	141.85	46.418	Zhang et al., 2015
39	Western Sakalin	Russia	141.811	46.541	Zhang et al., 2015
40	Western bay of Sakalin	Russia	142.059	47.141	Zhang et al., 2015
41	Northern Sakalin	Russia	141.92	48.843	Zhang et al., 2015
42	Gilak cape, Primorsky	Russia	138.55	46.983	Tatiana N Krupnova
43	Sosunova cape, Primorsky	Russia	138.33	46.533	Tatiana N Krupnova
44	Malaya Kema cape, Primorsky	Russia	137. 683	45.817	Tatiana N Krupnova
45	Amgu, Primorsky	Russia	137.15	45.4	Tatiana N Krupnova
46	Mosolova cape, Primorsky	Russia	136.567	45.083	Tatiana N Krupnova
47	Northern Primorsky	Russia	135.9	44.417	Tatiana N Krupnova

48	Eastern Primorsky	Russia	135.45	43.95	Tatiana N Krupnova
49	Vladivostok, Primorsky	Russia	131.87	43.115	Zhang et al., 2015

Reference:

Bi, Y., Hu, Y., & Zhou, Z. (2011). Genetic variation of *Laminaria japonica* (Phaeophyta) populations in china as revealed by RAPD markers. *Acta Oceanologica Sinica*, *30*(2), 103-112.

Gao, X., Endo, H., & Agatsuma, Y. (2015). Effect of increased seawater temperature on biomass, growth, and maturation of *Saccharina japonica*, near its southern limit in northern Japan. *Journal of Applied Phycology*, *27*(3), 1-8.

Yotsukura, N., Shimizu, T., Katayama, T., & Druehl, L. D. (2010). Mitochondrial DNA sequence variation of four *Saccharina*, species (Laminariales, Phaeophyceae) growing in Japan. *Journal of Applied Phycology*, *22*(3), 243-251.

Yotsukura, N., Maeda, T., Abe, T., Nakaoka, M., & Kawai, T. (2016). Genetic differences among varieties of *Saccharina japonica*, in northern Japan as determined by AFLP and SSR analyses. *Journal of Applied Phycology*,*28*(5) 1-13.

Zhang, J., Yao, J.T., Sun, Z.M., Fu, G., Galanin, D.A., Nagasato, C., Motomura, T., Hu, Z.M., and Duan, D.L. (2015). Phylogeographic data revealed shallow genetic structure in the kelp *Saccharina japonica* (Laminariales, Phaeophyta). *BMC Evolutionary Biology*, *15*, 237.

Table S5 Variable selection process for models with a beta-multiplier of 4. In step one, we compiled an initial MAXENT model (model 1) with all 26 variables and excluded 21 variables due to relative model contribution scores < 5%. In step two, the remaining set of variables was used to compile a new MAXENT model (model 2). We removed sstmax because of a contribution score < 5% and exclude sstmin due to correlation (0.9) with the variable of highest contribution (sstmean). The variables and their contributions in the model of highest performance (lowest AICc, model 3) are marked in yellow. Numbers in red indicate the exclusion of variables from one step to the next. AUC.Test, area under the receiver operating characteristic estimated from test data (maximum test AUC); AUC.Diff, the difference between AUC values estimated from test and training data.

				Model 1		Model 2		Model 3	
**Variable Short Form**	**Variable**	**Derivative**	**Unit**	**Contributions**	**Correlation coefficients**	**Contributions**	**Correlation coefficients**	**Contributions**	**Correlation coefficients**
**calcite**	Calcite	Mean	mol/m^3	0	-	-	-	-	-
**chlomax**	Chlorophyll	Maximum	mg/m^3	5.2314	0.888908	12.3418	-0.19653	12.1946	1
**chlomean**	Chlorophyll	Mean	mg/m^3	48.6527	1	43.5107	-0.16937	32.7935	0.888908
**chlomin**	Chlorophyll	Minimum	mg/m^3	0	-	-	-	-	-
**chlorange**	Chlorophyll	Range	mg/m^3	0	-	-	-	-	-
**damax**	Diffuse Attenuation	Maximum	m^{-1}	0	-	-	-	-	-
**damean**	Diffuse Attenuation	Mean	m^{-1}	0	-	-	-	-	-
**damin**	Diffuse Attenuation	Minimum	m^{-1}	0	-	-	-	-	-
**dissox**	Dissolved oxygen	Mean	ml/l	0	-	-	-	-	-
**nitrate**	Nitrate	Mean	µmol/l	0	-	-	-	-	-
**parmax**	Photosynthetically Active Radiation	Maximum	Einstein/m^2/day	0	-	-	-	-	-
**parmean**	Photosynthetically Active Radiation	Mean	Einstein/m^2/day	0	-	-	-	-	-
**ph**	pH	Mean	-	0	-	-	-	-	-
**phosphate**	Phosphate	Mean	µmol/l	4.0588	-	-	-	-	-
**Present.Surface.Ice.thickness.Lt.max**	Sea Ice Thickness	Longterm Maximum	m	0					
**Present.Surface.Ice.thickness.Lt.min**	Sea Ice Thickness	Longterm Minimum	m	0					
**Present.Surface.Ice.thickness.Max**	Sea Ice Thickness	Maximum	m	0					
**Present.Surface.Ice.thickness.Mean**	Sea Ice Thickness	Mean	m	0.2466					
**Present.Surface.Ice.thickness.Min**	Sea Ice Thickness	Minimum	m	0					

**Present.Surface.Ice.thickness.Range**	Sea Ice Thickness	Range	m	0					
**salinity**	Salinity	Mean	PSS	0	-	-	-	-	-
**silicate**	Silicate	Mean	µmol/l	0	-	-	-	-	-
**sstmax**	Sea Surface Temperature	Maximum	℃	2.5058	-	-	-	-	-
**sstmean**	Sea Surface Temperature	Mean	℃	29.4908	-0.16937	29.3111	1	55.0119	-0.19653
**sstmin**	Sea Surface Temperature	Minimum	℃	8.1101	-0.21689	14.8364	0.951569	-	-
**sstrange**	Sea Surface Temperature	Range	℃	1.7037	-	-	-	-	-
**Model Parameters**				10		10		8	
**AICc**				722.81		717. 46		703.24	
**AUC.Test**				0.81		0.77		0.82	
**AUC.Diff**				0.05		0.10		0.03	

Table S6 Haplotype distribution in each *Saccharina japonica* population. The haplotypes inferred from combined mtDNA (1890 bp) were identified by the capital H. Population codes in parentheses are the same as in Table 1 and Fig. S3.

Sampling locations	Combined mtDNA
Imabetsu, Aomori pref.(IM)	H1(4), H2(4)
Ishoya, Aomori pref.(IA)	H1(6), H2(3), H3(1)
Shiriya, Aomori pref.(SA)	H1(1), H2(3), H4(8), H5(1), H6(9), H7(1), H8(1), H9(2), H10(1)
Nemuro, Hokkaido (NE)	H11(12), H12(16)
Shiretoko Peninsula, Hokkaido (SP)	H12(6), H13(20), H14(6)
Wakkanai, Hokkaido (WA)	H14(23), H15(3), H16(1), H17(1), H18(1), H19(1), H20(1), H21(2)
Rebun, Hokkaid(RE)	H14(8), H22(1), H23(1)
Rishirifuji, Hokkaido (RI)	H14(20)
Rikibiru, Tomamae, Hokkaido (RT)	H12(23), H24(4), H25(1)
Hamamasu, Ishikari, Hokkaido (HI)	H12(7), H26(3)
Gokibiru, Ishikari, Hokkaido (GI)	H12(7), H26(1), H27(1)
Otaru, Hokkaido (OT)	H12(17), H28(3), H29(1)
Oshoro, Hokkaido (OS)	H12(16), H30(7), H31(1)
Yoichi, Hokkaido (YO)	H2(1), H12(6), H32(2), H33(1)
Kamui cape, Hokkaido (KA)	H12(20), H34(1), H35(1)
Usubetsu, Tomari, Hokkaido (UT)	H12(25), H36(1), H37(1), H38(1)
Fukushima, Hokkaido (FU)	H5(12), H12(4), H39(1), H40(1), H41(1)
Hakodate, Hokkaido (HA)	H12(12), H40(4), H42(9), H43(1), H44(1), H45(1)
Mori, Hokkaido (MR)	H12(20), H41(4), H47(1), H48(1), H461）
Oshamambe, Hokkaido (OH)	H1(2), H2(2), H12(3)
Kyrilian island (KY)	H2(1), H12(25), H48(1), H49(1)
Aniva Bay (west), Sakalin (AW)	H12(21), H33(1), H50(1), H51(1)
Aniva Bay (north), Sakalin (AN)	H12(26), H52(1), H53(2)
Shebunino, Aniva Bay, Sakalin (SH)	H12(3), H54(21), H55(1), H56(1), H57(1)
Western Sakalin (WS)	H12(5), H54(23), H55(1), H58(1)
Western bay of Sakalin (WB)	H54(14), H59(1), H60(1)
Northern Sakalin (NS)	H12(24), H61(1), H62(1)
Gilak cape, Primorsky (GK)	H54(21), H62(1), H63(1), H64(1), H65(1), H66(1)
Sosunova cape, Primorsky (SO)	H54(23), H67(1), H68(2), H69(1),
Amgu, Primorsky (AM)	H54(29), H70(1)
Mosolova cape, Primorsky (MO)	H54(9), H62(1), H71(6), H72(5), H73(1), H74(1), H75(1), H76(1)
Northern Primorsky (NP)	H54(8), H71(11), H72(4), H77(1)
Eastern Primorsky (EP)	H54(30)
Vladivostok, Primorsky (VL)	H12(3), H62(3)
Gangneung, Korea (GA)	H12(22), H30(1), H32(1), H78(3)

Table S7 Pairwise values of F_{ST} (below diagonal) and *P*-values (above) between 35*Saccharina japonica* populations based on microsatellites.

	IM	IA	SA	NE	SP	WA	RE	RI	RT	HI	GI	OT	OS	YO	KA	UT	FU	HA	MR	OH	KY	AW	AN	SH	WS	WB	NS	GK	SO	AM	MO	NP	EP	VL	GA
**IM**		NS	NS	***	***	***	***	***	***	***	***	***	***	***	***	***	***	***	***	***	**	***	***	***	***	***	***	***	***	***	***	***	***	***	***
**IA**	**0.014**		***	***	***	***	***	***	***	***	***	***	***	***	***	***	***	***	***	***	***	***	***	***	***	***	***	***	***	***	***	***	***	***	***
**SA**	0.020	0.026		***	***	***	***	***	***	***	***	***	***	***	***	***	***	***	***	***	***	***	***	***	***	***	***	***	***	***	***	***	***	***	***
**NE**	0.412	0.431	0.336		***	***	***	***	***	***	***	***	***	***	***	***	***	***	***	***	***	***	***	***	***	***	***	***	***	***	***	***	***	***	***
**SP**	0.260	0.270	0.202	0.331		***	***	***	***	***	***	***	***	***	***	***	***	***	***	***	***	***	***	***	***	***	***	***	***	***	***	***	***	***	***
**WA**	0.297	0.310	0.244	0.380	0.075		***	***	***	***	***	***	***	***	***	***	***	***	***	***	***	***	***	***	***	***	***	***	***	***	***	***	***	***	***
**RE**	0.230	0.242	0.180	0.428	0.230	0.226		***	***	***	***	***	***	***	***	***	***	***	***	***	***	***	***	***	***	***	***	***	***	***	***	***	***	***	***
**RI**	0.247	0.242	0.191	0.422	0.233	0.218	0.046		***	***	***	***	***	***	***	***	***	***	***	***	***	***	***	***	***	***	***	***	***	***	***	***	***	***	***
**RT**	0.271	0.278	0.212	0.361	0.202	0.224	0.282	0.266		***	NS	***	***	***	***	***	***	***	***	***	***	***	***	***	***	***	***	***	***	***	***	***	***	***	***
**HI**	0.235	0.255	0.190	0.345	0.161	0.179	0.262	0.252	0.020		NS	***	***	***	***	***	***	***	***	***	***	***	***	***	***	***	***	***	***	***	***	***	***	***	***
**GI**	0.252	0.259	0.197	0.374	0.198	0.193	0.276	0.259	0.012	0.006		***	***	***	***	***	***	***	***	***	***	***	***	***	***	***	***	***	***	***	***	***	***	***	***
**OT**	0.148	0.152	0.111	0.410	0.267	0.290	0.223	0.241	0.263	0.224	0.242		***	***	***	***	***	***	***	***	***	***	***	***	***	***	***	***	***	***	***	***	***	***	***
**OS**	0.209	0.204	0.158	0.353	0.188	0.197	0.201	0.200	0.140	0.102	0.100	0.152		***	***	***	***	***	***	***	***	***	***	***	***	***	***	***	***	***	***	***	***	***	***
**YO**	0.143	0.146	0.098	0.385	0.210	0.225	0.171	0.183	0.169	0.131	0.126	0.087	0.041		***	***	***	***	***	***	***	***	***	***	***	***	***	***	***	***	***	***	***	***	***
**KA**	0.161	0.161	0.125	0.370	0.220	0.219	0.196	0.204	0.161	0.119	0.126	0.091	0.060	0.046		***	***	***	***	***	***	***	***	***	***	***	***	***	***	***	***	***	***	***	***
**UT**	0.158	0.150	0.113	0.368	0.224	0.249	0.197	0.197	0.219	0.179	0.192	0.055	0.098	0.048	0.072		***	***	***	***	***	***	***	***	***	***	***	***	***	***	***	***	***	***	***
**FU**	0.059	0.072	0.031	0.323	0.190	0.231	0.172	0.188	0.190	0.161	0.171	0.116	0.131	0.069	0.110	0.104		***	***	***	***	***	***	***	***	***	***	***	***	***	***	***	***	***	***
**HA**	0.055	0.056	0.028	0.308	0.197	0.222	0.165	0.168	0.204	0.174	0.181	0.101	0.147	0.085	0.111	0.103	0.031		***	***	***	***	***	***	***	***	***	***	***	***	***	***	***	***	***
**MR**	0.175	0.172	0.142	0.444	0.302	0.316	0.277	0.280	0.272	0.251	0.253	0.178	0.216	0.158	0.166	0.147	0.143	0.140		***	***	***	***	***	***	***	***	***	***	***	***	***	***	***	***
**OH**	0.158	0.171	0.141	0.487	0.339	0.354	0.284	0.299	0.308	0.272	0.275	0.182	0.219	0.134	0.183	0.156	0.125	0.132	0.073		***	***	***	***	***	***	***	***	***	***	***	***	***	***	***
**KY**	0.244	0.256	0.189	0.246	0.246	0.282	0.211	0.234	0.234	0.232	0.226	0.272	0.221	0.207	0.230	0.239	0.183	0.171	0.258	0.273		***	***	***	***	***	***	***	***	***	***	***	***	***	***
**AW**	0.265	0.275	0.208	0.286	0.268	0.278	0.280	0.268	0.252	0.238	0.248	0.313	0.244	0.242	0.247	0.262	0.187	0.187	0.323	0.342	0.224		NS	***	***	NS	***	***	***	***	***	***	***	***	***
**AN**	0.243	0.252	0.194	0.278	0.257	0.256	0.257	0.248	0.238	0.221	0.228	0.295	0.226	0.220	0.225	0.243	0.173	0.175	0.314	0.323	0.218	0.007		***	***	***	NS	***	***	***	***	***	***	***	***
**SH**	0.277	0.258	0.206	0.390	0.276	0.287	0.241	0.176	0.265	0.262	0.275	0.295	0.244	0.254	0.240	0.244	0.211	0.187	0.313	0.350	0.260	0.155	0.155		NS	NS	***	***	***	***	***	***	***	***	***
**WS**	0.285	0.271	0.215	0.405	0.286	0.290	0.233	0.170	0.281	0.278	0.289	0.301	0.245	0.259	0.249	0.248	0.225	0.202	0.320	0.358	0.269	0.168	0.165	0.002		NS	***	***	***	***	***	***	***	***	***
**WB**	0.274	0.259	0.206	0.400	0.275	0.281	0.239	0.177	0.277	0.272	0.287	0.290	0.238	0.252	0.232	0.234	0.207	0.190	0.310	0.353	0.259	0.153	0.149	0.005	0.001		***	***	***	***	***	***	***	***	***
**NS**	0.263	0.268	0.210	0.291	0.270	0.273	0.273	0.254	0.257	0.245	0.249	0.314	0.248	0.245	0.246	0.260	0.189	0.189	0.325	0.343	0.220	0.007	0.008	0.137	0.154	0.134		***	***	***	***	***	***	***	***
**GK**	0.405	0.387	0.310	0.598	0.410	0.402	0.319	0.224	0.433	0.470	0.464	0.389	0.380	0.390	0.367	0.352	0.320	0.304	0.387	0.476	0.416	0.407	0.390	0.246	0.235	0.265	0.380		NS	NS	***	***	***	***	***
**SO**	0.407	0.389	0.315	0.599	0.410	0.407	0.331	0.234	0.440	0.477	0.470	0.395	0.385	0.397	0.374	0.356	0.325	0.307	0.395	0.486	0.421	0.415	0.399	0.251	0.239	0.271	0.387	0.002		NS	***	***	***	***	***
**AM**	0.401	0.384	0.307	0.590	0.407	0.403	0.309	0.214	0.430	0.466	0.460	0.389	0.377	0.389	0.367	0.350	0.320	0.302	0.396	0.478	0.405	0.399	0.381	0.223	0.210	0.242	0.369	-0.001	0.008		***	***	***	***	***
**MO**	0.417	0.401	0.315	0.604	0.430	0.431	0.352	0.255	0.453	0.488	0.485	0.393	0.389	0.400	0.375	0.354	0.320	0.307	0.411	0.496	0.425	0.421	0.407	0.266	0.256	0.297	0.391	0.059	0.057	0.045		*	***	***	***
**NP**	0.403	0.382	0.307	0.582	0.400	0.403	0.334	0.227	0.425	0.455	0.451	0.381	0.365	0.373	0.358	0.340	0.306	0.292	0.398	0.479	0.409	0.403	0.389	0.245	0.240	0.277	0.373	0.071	0.061	0.065	0.024		***	***	***
**EP**	0.414	0.396	0.321	0.598	0.417	0.416	0.355	0.245	0.446	0.479	0.475	0.392	0.388	0.397	0.378	0.354	0.327	0.310	0.415	0.494	0.430	0.425	0.408	0.254	0.246	0.282	0.394	0.050	0.036	0.048	0.048	0.038		***	***
**VL**	0.285	0.274	0.208	0.471	0.327	0.338	0.281	0.236	0.337	0.344	0.341	0.311	0.255	0.256	0.257	0.267	0.202	0.193	0.330	0.365	0.274	0.172	0.168	0.181	0.175	0.184	0.164	0.324	0.336	0.313	0.361	0.333	0.346		***
**GA**	0.154	0.164	0.108	0.324	0.196	0.206	0.188	0.208	0.174	0.137	0.143	0.138	0.095	0.070	0.106	0.123	0.094	0.094	0.174	0.155	0.175	0.229	0.210	0.255	0.262	0.256	0.238	0.386	0.395	0.386	0.397	0.378	0.399	0.257	

* $P < 0.05$; ** $P < 0.001$; *** $P < 0.0001$; NS, not significant.

Table S8 Pairwise values of F_{ST} (below diagonal) and *P*-values (above) between 35 *Saccharina japonica* populations based on mitochondrial DNA

	IM	IA	SA	NE	SP	WA	RE	RI	RT	HI	GI	OT	OS	YO	KA	UT	FU	HA	MR	OH	KY	AW	AN	SH	WS	WB	NS	GK	SO	AM	MO	NP	EP	VL	GA
**IM**		NS	NS	NS	***	***	***	***	***	***	***	***	***	***	***	***	***	**	***	***	**	***	***	***	***	***	***	***	**	***	***	***	***	***	***
**IA**	-0.035		**	NS	***	***	***	***	***	***	***	***	***	***	***	***	***	**	***	***	***	***	***	***	***	***	***	***	***	***	***	***	***	***	***
**SA**	0.239	0.298		**	***	***	***	***	***	***	***	***	***	***	***	***	***	***	***	***	***	***	***	***	***	***	***	***	***	***	***	***	***	***	***
**NE**	0.070	0.186	0.271		***	***	***	***	**	***	***	***	***	***	***	***	***	**	***	**	*	**	*	*	**	**	**	**	*	*	*	*	**	**	***
**SP**	0.683	0.712	0.636	0.477		***	***	***	***	***	***	***	***	***	***	***	***	***	***	***	***	***	***	***	***	***	***	***	***	***	***	**	***	***	***
**WA**	0.688	0.714	0.655	0.529	0.494		NS	NS	***	***	***	***	***	***	***	***	***	***	***	***	***	***	***	***	***	***	***	***	***	***	***	***	***	***	***
**RE**	0.889	0.884	0.719	0.799	0.676	0.027		NS	***	***	***	***	***	***	***	***	***	***	***	***	***	***	***	***	***	***	***	***	***	***	***	***	***	***	***
**RI**	0.719	0.739	0.633	0.562	0.565	0.021	0.075		**	***	***	***	***	***	***	***	***	***	***	***	***	***	***	***	***	***	***	***	***	***	***	***	***	***	***
**RT**	0.437	0.482	0.510	0.265	0.377	0.430	0.486	0.397		***	***	***	***	***	***	***	***	*	***	**	**	***	**	***	**	***	**	**	*	***	**	**	***	***	***
**HI**	0.777	0.792	0.683	0.630	0.634	0.665	0.860	0.754	0.443		NS	NS	*	*	*	***	***	*	*	***	***	***	***	***	***	***	***	***	***	***	***	***	***	***	***
**GI**	0.749	0.768	0.670	0.581	0.591	0.635	0.821	0.718	0.422	-0.012		NS	NS	NS	NS	***	***	*	*	***	***	***	***	***	***	***	***	***	***	***	***	***	***	***	***
**OT**	0.821	0.826	0.684	0.700	0.694	0.704	0.948	0.815	0.450	0.034	0.086		NS	NS	NS	*	**	***	NS	***	***	***	***	***	***	***	***	***	***	***	***	***	***	***	***
**OS**	0.803	0.813	0.704	0.685	0.687	0.705	0.885	0.786	0.487	0.041	0.090	0.001		***	***	***	***	***	***	***	***	***	***	***	***	***	***	***	***	***	***	***	***	***	***
**YO**	0.783	0.795	0.693	0.661	0.681	0.700	0.872	0.776	0.487	0.044	0.091	0.006	0.018		***	***	***	***	***	***	***	***	***	***	***	***	***	***	***	***	***	***	***	***	***
**KA**	0.900	0.897	0.752	0.822	0.761	0.766	0.980	0.894	0.533	0.063	0.132	0.021	0.016	0.023		***	***	***	NS	***	***	***	***	***	***	***	***	***	***	***	***	***	***	***	***
**UT**	0.683	0.708	0.659	0.553	0.621	0.647	0.761	0.662	0.466	0.216	0.234	0.192	0.205	0.206	0.269		NS	**	***	***	***	***	***	***	***	***	***	***	***	***	***	***	***	***	***
**FU**	0.761	0.777	0.701	0.657	0.697	0.709	0.843	0.748	0.516	0.422	0.424	0.427	0.419	0.412	0.530	0.018		***	***	***	***	***	***	***	***	***	***	***	***	***	***	***	***	***	***
**HA**	0.611	0.649	0.551	0.361	0.496	0.544	0.855	0.600	0.262	0.346	0.280	0.481	0.417	0.422	0.672	0.317	0.494		***	*	*	*	*	*	*	**	*	*	*	*	*	*	*	**	***
**MR**	0.918	0.912	0.759	0.849	0.774	0.777	1.000	0.915	0.539	0.073	0.145	0.042	0.020	0.027	0.000	0.281	0.554	0.732		***	***	***	***	***	***	***	***	***	***	***	***	***	***	***	***
**OH**	0.697	0.726	0.609	0.391	0.388	0.509	0.776	0.638	0.277	0.597	0.523	0.711	0.679	0.668	0.788	0.588	0.690	0.426	0.810		NS	*	NS	*	*	***	*	NS	NS	*	*	NS	*	*	***
KY	0.773	0.790	0.632	0.484	0.430	0.546	0.884	0.727	0.290	0.645	0.558	0.795	0.729	0.714	0.866	0.610	0.726	0.497	0.896	0.022		NS	NS	NS	NS	***	NS	NS	*	*	NS	NS	*	NS	***
**AW**	0.791	0.805	0.646	0.512	0.446	0.561	0.893	0.745	0.304	0.682	0.600	0.821	0.758	0.743	0.883	0.641	0.750	0.571	0.910	0.032	0.012		NS	NS	*	***	NS	NS	*	*	*	NS	*	*	***
**AN**	0.762	0.779	0.622	0.466	0.421	0.537	0.879	0.715	0.272	0.658	0.575	0.800	0.737	0.722	0.871	0.616	0.728	0.524	0.901	0.020	0.000	0.012		NS	*	***	NS	NS	NS	*	NS	NS	*	NS	***
**SH**	0.775	0.793	0.632	0.477	0.439	0.555	0.888	0.739	0.299	0.675	0.593	0.815	0.752	0.737	0.879	0.635	0.744	0.559	0.906	0.024	0.000	0.012	0.000		*	***	NS	NS	*	*	*	NS	*	NS	**
**WS**	0.705	0.733	0.611	0.408	0.403	0.518	0.791	0.649	0.285	0.610	0.537	0.724	0.689	0.678	0.801	0.593	0.696	0.424	0.824	0.074	0.073	0.092	0.076	0.084		***	*	*	*	**	*	NS	*	*	***
**WB**	0.684	0.711	0.610	0.476	0.524	0.578	0.795	0.651	0.337	0.662	0.616	0.731	0.713	0.704	0.813	0.615	0.703	0.505	0.832	0.433	0.502	0.522	0.489	0.513	0.438		***	***	**	***	***	**	***	***	***
**NS**	0.784	0.799	0.641	0.500	0.439	0.555	0.888	0.739	0.299	0.675	0.593	0.815	0.752	0.738	0.879	0.635	0.744	0.559	0.906	0.024	0.000	0.012	0.000	0.000	0.084	0.513		NS	*	*	NS	*	***	NS	***
**GK**	0.777	0.790	0.620	0.483	0.427	0.540	0.913	0.735	0.275	0.673	0.587	0.826	0.752	0.735	0.897	0.618	0.735	0.551	0.929	0.020	-0.001	0.012	-0.001	-0.001	0.080	0.502	-0.001		*	*	NS	NS	*	NS	***
**SO**	0.531	0.587	0.499	0.201	0.350	0.453	0.750	0.522	0.209	0.549	0.480	0.649	0.629	0.616	0.775	0.510	0.623	0.281	0.804	0.061	0.135	0.155	0.112	0.132	0.129	0.365	0.145	0.133		*	*	NS	NS	*	*
**AM**	0.616	0.654	0.581	0.346	0.397	0.493	0.689	0.559	0.264	0.555	0.499	0.630	0.626	0.619	0.717	0.555	0.643	0.365	0.734	0.143	0.211	0.227	0.203	0.219	0.208	0.411	0.219	0.206	0.169		*	NS	*	***	***
**MO**	0.726	0.748	0.603	0.421	0.405	0.518	0.849	0.676	0.267	0.632	0.553	0.767	0.713	0.699	0.847	0.594	0.706	0.472	0.876	0.048	0.050	0.063	0.047	0.052	0.095	0.461	0.052	0.052	0.119	0.197		NS	*	*	***
**NP**	0.650	0.681	0.536	0.306	0.355	0.467	0.885	0.612	0.195	0.602	0.515	0.760	0.689	0.671	0.872	0.532	0.662	0.365	0.914	0.013	0.028	0.045	0.023	0.032	0.059	0.399	0.032	0.034	0.048	0.141	0.044		NS	NS	*
**EP**	0.664	0.692	0.550	0.347	0.393	0.490	0.878	0.627	0.220	0.621	0.542	0.764	0.700	0.683	0.870	0.549	0.674	0.404	0.907	0.131	0.202	0.227	0.191	0.213	0.170	0.435	0.213	0.218	0.123	0.201	0.178	0.000		*	*
**VL**	0.750	0.771	0.631	0.460	0.425	0.540	0.841	0.699	0.296	0.646	0.569	0.773	0.724	0.711	0.841	0.619	0.724	0.507	0.866	0.056	0.053	0.066	0.051	0.056	0.105	0.486	0.056	0.055	0.142	0.218	0.083	0.055	0.195		***
**GA**	0.719	0.744	0.631	0.469	0.470	0.557	0.797	0.671	0.329	0.643	0.583	0.738	0.709	0.699	0.808	0.619	0.712	0.502	0.828	0.266	0.317	0.335	0.309	0.326	0.294	0.508	0.326	0.321	0.266	0.316	0.298	0.259	0.325	0.314	

* $P < 0.05$; ** $P < 0.001$; *** $P < 0.0001$; NS, not significant.

Table S9 Analysis of molecular variance (AMOVA) for different groups in *Saccharina japonica*

Source of variation	Degree of freedom	Sum of squares	Variance of components	Percentage variation	Φ-statistics
**(a) *Four BAPS groups based on mitochondrial DNA***					
Among groups	3	254.691	0.48	51.13	$\Phi_{CT} = 0.51^{***}$
Among populations within groups	31	101.194	0.13	14.38	$\Phi_{SC} = 0.29^{***}$
Within populations	740	238.668	0.32	34.50	$\Phi_{ST} = 0.66^{***}$
**(b) *Two STRUCTURE groups based on microsatellites***					
Among groups	1	1102.017	1.46	16.50	$\Phi_{CT} = 0.16^{***}$
Among populations within groups	33	2346.354	1.49	16.90	$\Phi_{SC} = 0.20^{***}$
Within populations	1501	8868.781	5.91	66.61	$\Phi_{ST} = 0.33^{***}$

***, $P < 0.001$

Table S10 Posterior parameter values for best model in stage 1 (Scenario 5) and stage 2 (Scenario 6) based on combined data. The unit of time is generations, and the generation time of *Saccharina japonica* is 2 years.

Parameter	Posterior parameter estimation		
	Median	Mode	95% credible interval
**Stage 1 (model 5)**			
N1: population size in the Hokkaido and Honshu (H)	64000	62500	38400-91800
N2: population size in Primorsky of Far-eastern Russia (P)	7230	7360	4870-9390
N3: admixture population size in Sakhalin of Far-eastern Russia (A)	35400	29400	15500-73500
NA: population size for ancestral populations	4960	4450	581-9480
t_2: time of divergence	10400	6830	3820-32700
t_1: time of admixture	3630	2820	939-8120
μ_1: mean mutation rate per generation for SSR	6.22×10^{-5}	6.25×10^{-5}	3.35×10^{-5}-9.02×10^{-5}
μ_2: mean mutation rate per generation for mitochondrial DNA	7.49×10^{-8}	7.61×10^{-8}	5.47×10^{-8}-9.49×10^{-8}
ra: admixture rate	0.595	0.595	0.286-0.887
**Stage 2 (model6)**			
N1: population size in the Hokkaido and Honshu (H)	63600	65900	39000-91400
N2: population size in Primorsky of Far-eastern Russia (P)	6910	6920	4780-9080
N3: admixture population size in Sakhalin of Far-eastern Russia (A)	34500	31900	15400-73200
NA: population size for ancestral populations	3310	1340	488-8940
t_2: time of pre-LGM divergence	13500	11300	10800-18900
t_1: time of populations admixture	3590	3340	832-8040
ra: admixture rate	0.577	0.595	0.203-0.901
μ_1: mean mutation rate per generation for SSR	7.09×10^{-5}	7.54×10^{-5}	4.28×10^{-5}-9.30×10^{-5}
μ_2: mean mutation rate per generation for mitochondrial DNA	7.38×10^{-8}	7.61×10^{-8}	5.39×10^{-8}-9.29×10^{-8}

U, uniform distribution;

Table S11 Morphological, ecological, and life history traits differences between *Saccharina japonica* in Russia and Japan

	Russia	Japan
Thallus color	Olive	Olive
Thallus length	2.0 – 3.5 m	1.5-3.0 m
Thallus width	0.20 – 0.35 m	0.05 – 0.35 m
Middle line	0.10 – 0.18 m	0.01-0.18 m
Rhizoid	Short, thick, fascicled	Short, thick, fascicled
Sporangia disposition on thallus	Sporangia are formed starting from the upper part of thallus	Sporangia are formed starting from the upper part of thallus
Sporulation period	July – October (up to November in Northern Primorye coast region)	October – December
Seedling	March – April (1 – 2 ^{o}C)	July – August (4 – 10°C)
Distribution pattern and preferred bottom	Wide distribution over all species areas (warm-temperate Far Eastern waters); it grows along shore (coast-wisely); rocky bottom; exposed coast; open entrance capes	rocky bottom along coast of Hokkaido Island and northern coast of Honshu Island
Duration of life history	1 or 2 years	2 or 3 years

Reference:

Evgeniy, S. B., Krupnova, T.N., & Ayala, F.J., (2012). DNA variation in the phenotypically-diverse brown alga *Saccharina japonica*. *BMC Plant Biology*, *12*(1), 108-108.

Kawashima, S., (2012) Morphological and Taxonomy of the Laminariaceous Algae in Cold Water Area of Japan (In Japanese), Oya Nisan Publisher.
